# Monitoring intrapartum fetal heart rates by mothers in labour in two public hospitals: an initiative to improve maternal and neonatal healthcare in Liberia

**DOI:** 10.1186/s12884-020-02921-z

**Published:** 2020-06-15

**Authors:** K. Borzie, N. Jasper, D. P. Southall, R. MacDonald, A. A. Kola, O. Dolo, A. Magnus, S. D. Watson, M. Casement, B. Dahn, W. Jallah

**Affiliations:** 1 CB Dunbar Hospital, Gbarnga, Liberia; 2 Fish Town Hospital, Fish Town, Liberia; 3 Maternal and Childhealth Advocacy International (MCAI), 1 Columba Court, Laide, Highland, IV22 2NL Scotland; 4 MCAI, Laide, Scotland; 5 MCAI, CB Dunbar Hospital, Gbarnga, Liberia; 6 Monrovia, Liberia; 7 CH Rennie Hospital, Kakata, Liberia; 8 The Royal Gwent Hospital, Newport, UK; 9 MCAI, Belfast, UK; 10 University of Liberia, Monrovia, Liberia; 11 Ministry of Health and Social Welfare, Monrovia, Liberia

**Keywords:** Stillbirth, Fetal monitoring, Birth asphyxia, Low-resource setting

## Abstract

**Background:**

In low-resource settings with few health workers, Fetal Heart Rate (FHR) monitoring in labour can be inconsistent and unreliable. An initiative to improve fetal monitoring was implemented in two public hospitals in rural Liberia; the country with the second lowest number of midwives and nurses in the world (1.007 per 10,000 of the population). The initiative assessed the feasibility of educating women in labour to monitor their own FHR and alert a midwife of changes detected.

**Methods:**

Four hundred seventy-four women admitted in labour without obstetric complications were approached. Four hundred sixty-one consented to participate (97%) and 13 declined. Those consenting were trained to monitor their FHR using a sonicaid for approximately 1 minute immediately following the end of every uterine contraction and to inform a midwife of changes. If changes were confirmed, standard clinical interventions for fetal distress (lateral tilt, intravenous fluids and oxygen) were undertaken and, when appropriate, accelerated delivery by vacuum or Caesarean section. Participants provided views on their experiences; subsequently categorized into themes. Neonatal outcomes regarding survival, need for resuscitation, presence of birth asphyxia, and treatment were recorded.

**Results:**

Four hundred sixty-one out of 474 women gave consent, of whom 431 of 461 (93%) completed the monitoring themselves. Three hundred eighty-seven of 400 women who gave comments, reported positive and 13 negative experiences. FHR changes were reported in 28 participants and confirmed in 26. Twenty-four of these 26 FHR changes were first identified by mothers. Fetal death was identified on admission during training in one mother. Thirteen neonates required resuscitation, with 12 admitted to the neonatal unit. One developed temporary seizures suggesting birth asphyxia. All 26 neonates were discharged home apparently well. In 2 mothers, previously unrecognized obstetric complications (cord prolapse and Bandl’s ring with obstructed labour) accompanied FHR changes. Resuscitation was needed in 8 neonates without identified FHR changes including one of birth weight 1.3 Kg who could not be resuscitated. There were no intrapartum stillbirths in participants.

**Conclusions:**

Women in labour were able to monitor and detect changes in their FHR. Most found the experience beneficial. The absence of intrapartum stillbirths after admission and the low rate of poor neonatal outcomes are promising and warrant further investigation.

## Background

The need to improve identification and management of intrapartum placental insufficiency and fetal distress is particularly relevant in low-income countries, such as Liberia. Yet it is in such settings that the methods and means to do so are severely limited by the lack of health workers and by material poverty. Challenges include the lack of suitably skilled birth attendants, particularly in rural settings; the high workload with each attendant often having to care for several women in active labour, especially during the night; and the lack of resources to fund, maintain and interpret fetal monitoring devices. As quoted by *The Lancet* Every Newborn Study group: *“Sensitive, specific, and simpler methods for detection of fetal compromise during labour could have a major effect on intrapartum stillbirths and early neonatal deaths, as long as linked with emergency obstetric care”* [1]*.*

The scale of the shortage of suitably trained health workers, such as nurses and midwives to undertake any fetal monitoring is highlighted in Table 1, which shows the numbers of nurses and midwives per 10,000 of the population as determined by WHO in 2019 in its Global Health Observatory Data Repository [2]. Seventeen out of 194 countries had < 5.0 midwives and nurses per 10,000 of the population and 15 out of 194 countries had > 100 midwives and nurses per 10,000 of the population.

**Table 1:** Lowest and highest numbers of nurses and midwives per 10,000 of the population in 194 countries in the world [2]

17 Countries with lowest numbers of midwives and nurses	Last Date of Data recorded	No. midwives and nurses per 10,000 population	15 Countries with highest numbers of midwives and nurses	Last Date of Data Recorded	No. midwives and nurses per 10,000 population
Somalia	2014	0.611	Norway	2017	181.247
**Liberia**	**2015**	**1.007**	Switzerland	2016	172.828
Madagascar	2014	1.059	Iceland	2017	156.806
Central African Republic	2015	2.039	Finland	2016	147.230
Malawi	2016	2.528	Republic of Ireland	2016	142.949
Togo	2015	2.980	Germany	2016	131.967
Bangladesh	2017	3.067	Luxembourg	2017	123.496
Dominican Republic	2017	3.099	Australia	2016	126.612
Niger	2014	3.109	Uzbekistan	2014	120.739
Senegal	2016	3.129	Sweden	2016	115.434
Afghanistan	2014	3.200	Japan	2016	115.184
Chad	2016	3.637	Belarus	2014	114.383
Mali	2016	3.820	Belgium	2016	111.011
Guinea	2016	3.844	New Zealand	2017	109.550
United Republic of Tanzania	2014	4.126	Denmark	2016	103.004
Mozambique	2017	4.436			
Democratic Republic Congo (DRC)	2013	4.700		Monaco (Pop.38,000) and Niue (Pop. 1600) excluded having high proportions but very small populations	

The two countries with the lowest numbers of midwives and nurses currently recorded by WHO are Somalia and Liberia. Both countries have experienced recent armed conflict. Between 2014 and 2015, Liberia also experienced an Ebola outbreak, which killed many healthcare staff. Unfortunately, since recovery from Ebola, the international community’s statements that they would improve the public health infrastructure of Liberia have not materialized [3].

Table 1 also shows that high-income countries have more than 100 times the numbers of midwives and nurses than Liberia, available to attend pregnant women and their babies.

Moreover, as a result of increasing inflation in Liberia, (7.4% in 2009 to 22.3% in 2019) [4] and worsening poverty, the employment of midwives in the public hospitals has reduced, their salaries have decreased, and sometimes, they may not be paid for several consecutive months. Consequently, their morale has fallen, and partographs are infrequently completed according to WHO recommendations. Although the Ministry of Health has made great efforts to educate midwives to recommended international standards [5], the lack of funds available to the national health service facilities, frequent absence of essential drugs, medical and surgical supplies, together with a lack of international funding, make providing high quality health care difficult to achieve, especially in rural areas where attracting health workers of all levels is difficult.

Labour wards in rural Liberian hospitals are frequently over-crowded with patients and it is unusual for any mother in labour to be accompanied by a relative or for them to receive the levels of attention and care, both to themselves and to their unborn babies, which are essential to prevent and / or treat intrapartum complications.

A study conducted in 2010 in a Liberian referral hospital showed that out of 1656 deliveries in 1 year, there were 196 perinatal deaths; 143 classified as stillbirth and 53 classified as early neonatal death [6]. The majority of stillbirths (56.6%) presented as antenatal stillbirths with no fetal heart sounds documented on admission. Thirty-seven (25.9%) had documented fetal heart rates upon admission with the stillbirth occurring during the intrapartum period. There was no documentation of presence or absence of fetal heart rates in 25 of the stillbirth records (17.5%). Thirty-one percent of cases had no maternal or obstetrical diagnosis recorded in the chart when a stillbirth occurred. Of the 53 early neonatal deaths, 47.2% occurred on the first day of life. The largest single contributor to early neonatal death in the sample was birth asphyxia identified by poor Apgar scores (< 4 at 5 min). Seventeen of the 53 early neonatal deaths (32%) were due to birth asphyxia [6].

After this health improvement initiative / feasibility study began in July 2017, in February 2018, WHO produced an updated guideline concerning intermittent FHR auscultation during labour using either a Doppler ultrasound device or a Pinard fetal stethoscope [7] This WHO expert group reported that monitoring of the fetal heart rate during labour was inadequate in many low- and middle-income country (LMIC) settings, and that this problem needed to be strongly addressed through quality improvement initiatives in these settings. A review of qualitative studies exploring women’s experiences of labour and childbirth, suggested that women would prefer a more hands-on, woman-centred approach to care and were likely to favour any technique that allows for this approach, [8] WHO have also pointed out that Doppler monitoring allows a woman to hear the fetal heartbeat, which provides reassurance and could add to its appeal over Pinard auscultation. WHO’s 2015 State of inequality report [9], indicates that women who are poor, least educated, and residing in rural and remote areas have lower access to healthcare intervention coverage than more advantaged women. The 2018 WHO guideline [7] also emphasised studies reporting that adequate monitoring of labour progress is often lacking in such settings, and that the FHR may only rarely be auscultated [10, 11].

Such inadequate FHR monitoring represented the situation in rural public hospitals in Liberia in 2017, which led to the design and implementation of this present initiative.

Globally, intrapartum-related complications are reported to cause an annual 1.2 million stillbirths, 700,000 term newborn deaths, and an estimated 1.2 million newborn babies developing neonatal encephalopathy (birth asphyxia) with 233,000 survivors developing moderate or severe neurodevelopmental impairment [12]. The countries with the highest stillbirth and neonatal mortality rates are in Sub-Saharan Africa [1], where between 25.1 and 34.2 stillbirths occurring for every 1000 births, with an estimated 51% of these deaths happening intrapartum [13]. For example, in a rural hospital in Tanzania, the stillbirth rate was 27/1000 live births, with 16/1000 occurring intrapartum [14] and 27% of deaths in the first 6 days of life related to intrapartum causes.

Compared with well-resourced countries, intrapartum-related neonatal mortality rates are 25-fold higher and intrapartum stillbirth rates up to 50 times higher in the lowest-income countries [15] where rehabilitation services for children with neuro-developmental impairments are poor or absent.

The integration of obstetric and neonatal care to manage complications for both mother and fetus during labour [16] is critically important with task-sharing being particularly relevant in low resource settings. To the best of our knowledge, mothers have not previously participated in FHR monitoring during labour. Their contribution (as a form of task-sharing) could be valuable for the wellbeing of both themselves and their babies and could be of assistance to overworked health care professionals who are too busy to undertake regular fetal monitoring. WHO recommendations for intrapartum fetal monitoring by midwives may be possible to achieve in well-resourced countries (100–180 nurses and midwives per 10,000 population [2]) but may be aspirational in poorly resourced countries, such as Liberia, which has 1.007 nurses and midwives per 10,000 population [2].) As part of a maternal and neonatal health care improvement initiative, we assessed whether maternal participation in monitoring the FHR during labour is a potentially feasible, effective, and sustainable approach to improving intrapartum and neonatal survival in situations with few and overworked skilled birth attendants.

## Methods

We have used and adhered to the revised standards for quality improvement reporting excellence (SQUIRE 2.0) to report this initiative [17].

Given the major shortage of midwives in Liberia, the first aim of this initiative, was to assess the feasibility of educating women in labour to assess their FHRs with a portable doppler monitor and to alert the midwife if they detected changes in the FHR which might indicate fetal distress.

The second aim of this initiative was to facilitate system changes to educate and enable the attending midwife, who would likely be caring for several individual women in labour, to initiate an immediate remedial course of action should she/he be alerted by a woman in labour who detected changes in her FHR. The questions posed, and the measured outcomes of this intervention, are shown in Table 2.

**Table 2:** Questions to be addressed by this initiative

Questions to be addressed	Outcomes
1) Can women in labour be educated to detect changes in FHR (especially fetal bradycardia and fetal tachycardia) through self-monitoring of the FHR with a doppler monitor and alert the attending midwife?	1a) Number of women (out of all approached for consent) in two hospital maternity units willing to participate in the self-monitoring initiative 1b) Number of women (out of all approached for consent) participating in the self-monitoring initiative who were able to detect FHR changes which were confirmed as abnormal and possibly harmful by the clinically attending midwife.
2) Do attending midwives respond to alerts from women in labour regarding suspected FHR changes and do they initiate the agreed course of action (the birth asphyxia and stillbirth prevention protocol) every time in a timely manner?	2a) Number of times (out of all possible) an attending midwife responded to an alert from a participating woman in labour who self-detected a potentially harmful change in FHR. 2b) Action taken by an attending midwife responding to an alert by a woman in labour of a possible FHR change and whether the agreed birth asphyxia and stillbirth prevention protocol had been followed.
3) Did the labouring women find the experience of monitoring their unborn babies helpful?	3a) How many mothers found the monitoring helpful? 3b) How many mothers found the monitoring difficult? 3c) How many mothers had to discontinue the monitoring?
4) What measure could be implemented to improve the attainments of the above first three objectives and result in a sustainable programme?	4a) Improvements in obtaining consent 4b) Improvements in the documentation of changes in FHR 4c) Feedback of results to the midwives on the maternity wards 4d) How to achieve sustainability given the temporary availability of trainee obstetric clinicians
5) Was the attending midwife able to initiate an immediate course of treatment when she was alerted by a woman in labour who had identified changes in her FHR? 6) Were professionals trained in advanced obstetrics and neonatal care able to provide effective treatment when asked to by the attending midwife?	Treatments given and outcomes in the mother Treatments given and outcomes in the newborn infant

### Setting

In Liberia, over the past 6 years, a partnership between the Ministry of Health and Social Welfare (MOHSW), The World Health Organization (WHO), The United Nations Population Fund (UNFPA), the Liberian Board for Nursing and Midwifery (LBNM) and the registered charity (not-for profit organization), Maternal and Childhealth Advocacy International (known as MCAI), has been established to train senior birth attendants (19 midwives and 2 physician assistants) in advanced obstetrics to become obstetric clinicians, able to undertake emergency clinical procedures to expedite the delivery of a distressed fetus, such as vacuum delivery and Caesarean section [18]. Currently, 10 obstetric clinicians have completed training, 10 more are currently in their final year of training and 9 more have just been appointed following an examination and interview.

More recently, the partnership initiated a new programme to improve neonatal resuscitation and hospital neonatal care by training selected nurses and midwives to become advanced neonatal nurse practitioners (neonatal clinicians) and is now based in both of the two hospitals involved. Currently three have completed their training, 6 are in their final year and 8 have just been appointed following an examination and interview.

The fetal monitoring initiative took place in CB Dunbar Hospital in Bong County and CH Rennie Hospital in Margibi County. The main training hospital for both obstetric clinicians and advanced neonatal nurse practitioners (neonatal clinicians) is CB Dunbar Maternity Hospital. During this initiative and working in these two hospitals, there was one fully qualified and licensed obstetric clinician, 4 trainee obstetric clinicians and 9 trainee neonatal clinicians at CB Dunbar Hospital. There were 5 trainee obstetric clinicians at CH Rennie Hospital but no neonatal clinicians at the time of this feasibility study.

### Management committee for this health care improvement initiative

This initiative represented an integrated project between obstetrics and neonatology. Both qualified and trainee obstetric clinicians and trainee neonatal clinicians and their trainers were involved.

The management committee oversaw the day to day running of the initiative and was responsible for the data analysis. If there were any problems identified, in particular involving the need for improving the recording methods, the management committee would make the necessary changes to the initiative as appropriate.

As members of the management committee, two trainee Obstetric Clinicians (KP and NJ), and the international advanced neonatal nurse practitioner (AK) managing the recently established neonatal intensive care unit, led the day to day running of the fetal monitoring component of this initiative.

The fetal monitoring project started as a service delivery intervention; when authors realized the possible significance of the outcomes, we decided to approach the work from a research angle for publication. As a programmatic intervention, it was fully within the domain of the Ministry of Health to implement. Re-framed as a research project, it was relevant to seek National Research Ethics Board approval.

Because of poverty and complex social circumstances, it is common for mothers in Liberia to be aged under 16 years. Also, young mothers frequently attend the hospitals in labour by themselves, with no legally assigned family members in attendance and sometimes only a traditional birth attendant for support. Many women and adolescent girls do not know their age, and the illiteracy rate in Liberia is high (63%) [19].

### Participants

Over a 15-month period (from 31 July 2017 to 24 October 2018), 474 women admitted in the active first stage or second stages of labour, who were not experiencing any complications such as haemorrhage, severe pre-eclampsia or obstructed labour, were invited to participate. Before participation, each mother was asked to give her informed consent by a trainee or fully trained obstetric clinician. Initially (given a female literacy rate from UNICEF data 2011–2016 [19] of those aged 15 to 24 years of approximately 37%) this consent was requested verbally and recorded and later (after 27th April 2018) a dedicated consent form was signed (either by fingerprint or in writing).

From the start, all authors considered this work as the implementation of a new project that would improve health service delivery, in particular regarding fetal monitoring during labour. The severe lack of midwives and the infrequency of adequate completion of the partograph, indicated to the authors that this project was an essential part of health service delivery rather than a research study. The project had the prior approval of the Liberian Ministry of Health. As the focus was improved health service delivery and did not involve any invasive procedures, authors initially sought verbal consent from all potential participants. However, as the initial results of the project were so encouraging, authors recognised the value in sharing our experience of this project through a peer-reviewed publication and so changed the consent process to require written consent from all participants. Informed consent, written or verbal, was therefore obtained from all participants.

Throughout, this non-invasive project was supervised by the Liberian Ministry of Health (the original Minister of Health, BD, up to January 2018 and current Minister, WJ, from January 2018 are both authors of this paper).

The seeking of consent, whether verbal or written, was dependent on each woman understanding what was being requested of her. The consent form (part of the Additional File 1) was read out and discussed with each woman before she was enrolled in the study. Only women or adolescent girls (those potential participants aged under 18 years) who fully understood what they were being asked to undertake were recruited. The obstetric clinicians who gained consent were skilled health workers with years of experience in managing pregnant women in Liberian public hospitals. All had undergone a medical ethics and professional standards course as part of their training. Therefore, these health workers were well placed to ascertain if each woman who was invited to participate understood what was required. Each woman could change her mind at any time, and her wishes were always respected.

### Plan of investigation

Between contractions, each participating woman was shown and educated by an obstetric clinician in how to use a re-chargeable, battery operated, fetal doppler heart sound monitoring device (a sonicaid), including the best position to place the probe on the mother’s abdomen. This training usually lasted between 10 and 15 min. The mother was also educated in what was a normal heart rate and what was slow or fast, by tapping out a rhythm. Mothers were asked to monitor for approximately a 60 s period immediately following the end of *every* contraction. However, it wasn’t, in the authors’ opinion, of crucial importance or appropriate to insist that participating women monitor for exactly 60 s, which may be difficult. In addition, it is/was the beginning of the 60 s period that we considered of most clinical relevance to detecting changes in the FHR that might indicate fetal distress.

Having determined what was a normal rate, the mother was then asked to identify and immediately inform a midwife of any changes in the FHR that she detected. In the ward areas in the hospitals undertaking this initiative, there is no buzzer or bell alert and so if a participating woman thought that she had detected a change in FHR, she called out for a midwife or obstetric clinician to come and confirm possible changes. As the labour wards in both hospitals were small, this system worked well. Sometimes, the mothers reported actual heart rates (if literate) but mostly identified a fall or increase in heart rates without counting the numbers involved. The midwives and obstetric clinicians who responded to the mother, recorded and reported the actual heart rates. Additional File 1 contains the form that was used for the recording process which was completed as soon as possible after delivery.

Additional file 2 is a Table containing the comments made on the monitoring process by the mother, either written directly or transcribed for illiterate mothers by the obstetric clinician completing the form. To help with anonymity, age groups for each participant have been used instead of actual ages.

In all participating women, the partograph was supposed to be completed according to WHO recommendations by a midwife on the labour ward every 30 min during the first stage and every 5 min during the second stage of labour [20, 21]. However, in reality, the fetal section of the partograph was inconsistent and rarely completed due to time pressures on the small number of midwives available.

If a participating woman decided that she could not continue to monitor the FHR, for whatever reason, her wishes were respected and documented. If a woman’s condition during labour made it difficult for her to continue making recordings, a midwife or obstetric clinician would, if possible, and if time allowed, assist or take over the monitoring from her with the woman’s permission.

If a woman declined to undertake the monitoring herself but still wanted her fetus to be monitored, with the mother’s permission, the monitoring could be done by a midwife or by an obstetric clinician, provided they had time available given their heavy workloads.

### Actions by midwives and/or obstetric clinicians

The midwives working in the two hospitals were informed of the plans and scope of the initiative by the management committee team and educated in their role and responsibilities regarding the initiative, especially that they must, if possible, respond immediately when alerted by the participating women to a change in FHR and to examine the woman. If the FHR was potentially of concern, the midwife would then immediately notify the obstetric clinician and/or doctor on duty who would assist the midwife in the management of labour until the FHR had either recovered or the baby was safely delivered (as per the birth asphyxia prevention protocol: see below). This remedial protocol was already in place on the maternity units should potential fetal distress be detected. However, unfortunately, FHR monitoring as part of the partograph was rarely undertaken prior to this initiative for reasons outlined in the earlier “Background” to this project. At CB Dunbar Hospital, the neonatal clinician on duty would also be asked to be present at the delivery to resuscitate the neonate if necessary.

### The birth asphyxia prevention protocol

1. if a participating woman in labour detected a possible FHR change (bradycardia or tachycardia) she immediately notified an available midwife.
2. The midwife checked the FHR and the mother’s condition.
3. If a bradycardia (< 120 beats/minute) or tachycardia (> 160 beats/minute) were present, the midwife immediately notified the obstetric clinician or doctor on duty.
4. If the FHR was normal, the midwife immediately began a period of continuous fetal monitoring including the time up to the next contraction. She / he also listened to the FHR during the whole of the next contraction and immediately following that contraction.
5. If a suspicious FHR was detected during the 1 minute following the next contraction, the midwife took the following actions:

(i) ensured the mother was not lying flat on her back by providing left lateral tilt, examined the liquor if membranes had ruptured, and noted whether meconium staining of amniotic fluid was present.
(ii) ensured that the obstetric clinician and/or doctor on duty was present.
(iii) provided additional inspired oxygen (when available), secured an intravenous cannula and gave a bolus of either 0.9% saline or Ringer Lactate solution and where there was suspicion of ketosis, added a bolus of 50% dextrose to the IV infusion [21].

6. If there was evidence of fetal distress (late decelerations, persistent bradycardia, persistent tachycardia, meconium stained liquor) then the mother was assessed by the obstetric clinician or doctor to manage any maternal obstetric problem that could be responsible for the fetal bradycardia/tachycardia and assess for urgent immediate delivery as follows:

(i) If the cervix was fully dilated and there were no contraindications, a vacuum delivery was undertaken.
(ii) if the cervix was not fully dilated, then the woman was prepared for an emergency Caesarean section (CS) and taken to the operating theatre where she was re-examined. If the fetus was still alive and the cervix still not fully dilated, a CS was performed. An abdominal ultrasound scan was often helpful at this time, not only to check whether the fetus was still alive, but also related to other clinically relevant issues, such as to determine the lie and position of the fetus and confirm the position of the placenta.

7. Under either circumstance outlined in 6 above, a neonatal clinician or midwife experienced in neonatal resuscitation, was immediately made available for when the baby is delivered. She/he would ensure that the equipment needed for resuscitation was available and functioning.

### The outcome for the mother and baby

The clinical condition of the baby at birth, the need for neonatal resuscitation, admission to the neonatal unit and any signs of subsequent birth asphyxia (also known as Hypoxic Ischaemic Encephalopathy: HIE) were documented. A clinical summary of the pregnancy and delivery was also documented.

### The views of the mothers on the fetal monitoring process

From 23rd October 2017, through written or verbal comments (the latter transcribed by the midwife or obstetric clinician), mothers were asked for their views on the monitoring process.

One hundred fifty-three out of 154 participants who had consented at CH Rennie hospital (where the project started on 21st March 2018) were approached for comment (on admission one potential participant’s fetus was found to be dead during her training in monitoring).

This request for comments was not in place for the 33 of the consented mothers, who had enrolled before 23rd September 2017 at CB Dunbar Hospital.

Two authors (DS and RM), categorized the comments retrospectively by first agreeing the common themes after reading all of the comments provided by the participating women. Any discrepancies between DS and RM were discussed and agreed by consensus.

The comments in Table 3 were selected as being representative of the basic themes, as subjectively agreed by the two authors RM and DS. The full set of comments from all participants are available in the Supplementary Information (Additional file 2).

**Table 3:** Selected maternal comments from participants from CB Dunbar and CH Rennie Hospitals

Age group (years)	Comment
N/A	The monitoring was fine, it gave me courage to go through my pain knowing my baby was fine
N/A	Mother express her interest in measuring her baby FHR. She further stated due to the exercise she will always come to NAME OF HOSPITAL for maternity care during pregnancy
N/A	I felt that I am important when you told me to be a part of my baby monitoring process. It helps me a lot
29–39	The monitoring was good. It help even us that cannot read or write listen to our own baby
N/A	I thank God for the programme I am happy to hear my baby heart-beat. Please continue it
29–39	The monitoring was good, it helps me give the power to push my baby
18–28	Listening to my baby heart sound was very helpful to me. I felt that my right was respected as I took in my baby monitoring. Thanks for this program. I am happy.
18–28	I am happy to hear my baby heart. I knew that I was carrying a live baby in my womb.
17 and below	Thank you for this. It help me but I was in pain and so it make me angry first but I overcome it later
29–39	Getting involved in the process is something amazing to me. I felt part of my care and thank God that I have a live baby.
18–28	I feel important in the coming of my baby. This modern method is very important it help a lot thank you
18–28	I like it so much doctor that real good thing the government put in place here. I will tell all my sisters that pregnant to come to the hospital
29–39	According to mum it is a good step to do because it helps you to notice danger sooner.
N/A	Mother felt comfortable using this method. She told me that she will encourage her friends, who have not been seeking care at NAME OF HOSPITAL, because of the fetal monitoring The only problem was locating the FHT on her abdomen
18–28	I felt good listening to my baby it helped me to learn a new thing
18–28	According to mum, this is the first time seeing patient to be working for herself. She said it is a good thing to do but when in labour is bad because of the pain.
18–28	Patient said she’s very happy because she seen baby breathing well and she herself okay. According to patient any time she pregnant she will come and give birth to NAME OF HOSPITAL.
29–39	According to mum, she love the procedure but is not easy to go through.
29–39	According to mum, she love the idea because other pregnant women goes to the hospital and comes back with no baby in their hands it looks sorryfull.
29–39	Patient is educated. Getting involved in the process is something amazing to me. I felt part of my care and thank God that I have a live baby
17 and below	Patient admitted that it was good thing for herself to listen to her baby heart-beat. It made her believe that her baby can breathe inside her mother’s womb.
18–28	Thank you so much for your patience but at least I able to hear my baby. My last belly I don’t see someone doing it for me
18–28	According to patient she was surprised to know that baby heart can beat in the mother stomach and it help her to know about her baby wellbeing.
18–28	I appreciate hearing my baby until I born my baby. I like to have the same chance to listen to my unborn baby the next time I am in labour
29–39	I enjoy listening to my baby but my next labour there should be pain medicine for labour
N/A	The monitoring was alright for me. It help me to put more effort for my baby. To know that my baby is still living in my stomach.
29–39	It is hard to be in pain and monitor your baby. You must be doing it for us. Thank God my baby is living but it is too hard. The machine can cause more pain on the stomach.
29–39	It help me because it did not allow me to go to surgery. It help me because my baby was born alive and by normal vaginal delivery. It help me so much even though is more difficult to do but I try doing to have got good result. (vaginal breech delivery).
18–28	It is very good and helpful to me. At least all “big belays” should know how to do the monitoring before the stomach can hurt.
29–39	I like the monitoring it make my baby live. No problem with the monitoring. It only hard to hold the machine when your stomach hurting.
18–28	The monitoring was good make me feel close to my baby
17 and below	Patient said while doing she felt bad but after she gave birth she felt fine because it helps you to know the danger sign and good sign about your baby
29–39	I appreciate the process of monitoring my baby until I born. It was good for me because I stay long in labour but I was still hearing my baby which made me happier
N/A	The monitoring was alright for me. It help me to put more effort for my baby. To know that my baby is still living in my stomach.
18–28	Thank you very much the monitoring help me to know that I was carrying a baby whose heart was beating. It was my first time to know
17 and below	The monitoring help me to know that my babies are two in my stomach. I tell the programme thank you for coming to us

Additional maternal comments are reported in Tables 4, 5 and 6 below and in the Additional File 2. Abbreviations are defined in the list given earlier in the manuscript

### Data analysis

MCAI was responsible for the data analysis and undertook a descriptive analysis.

**Fig. 1:**
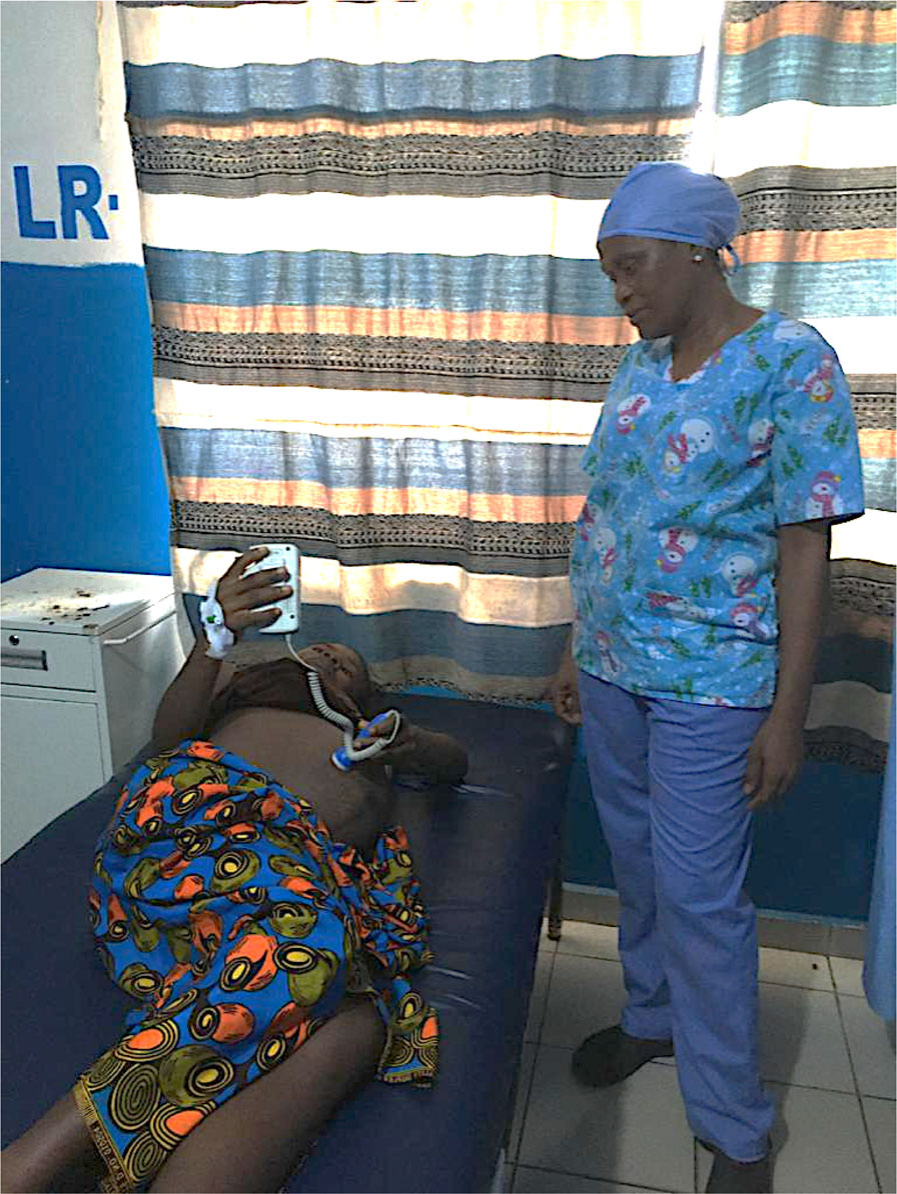
One of the mothers at CB Dunbar Hospital checking her unborn baby’s heart rate during the first stage of her labour immediately following a uterine contraction using a sonicaid (fetal doppler battery operated machine). Written consent to use this picture in this publication was obtained from the participant and is available on request

The lead obstetric clinician in each hospital (KP and NJ) was responsible for charging the batteries and for ensuring gel, consent, and monitoring forms were constantly available. Forms were scanned and sent to the UK by MCAI programme and finance managers in Liberia (Fig. 1).

## Results

**Fig. 2:**
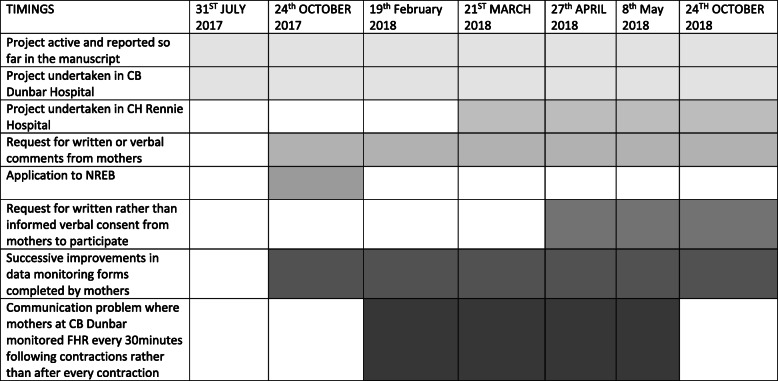
A Flow chart which describes the timelines of the various interventions undertaken up to the completion of this manuscript. The project meanwhile at the time of this publication, continues in both hospitals

The project meanwhile, at the time of this publication, continues in both hospitals (see Fig. 2).

### Consent and monitoring (see flow chart Fig. 3)

Over 15 months from 31 July 2017 until 24 October 2018, 474 women (157 from CH Rennie and 317 from CB Dunbar Hospitals) admitted in the first or second stages of labour and without obstetric complications were approached. Four hundred sixty-one gave their informed consent to participate in the FHR monitoring (97%) and 13 declined.

**Fig. 3:**
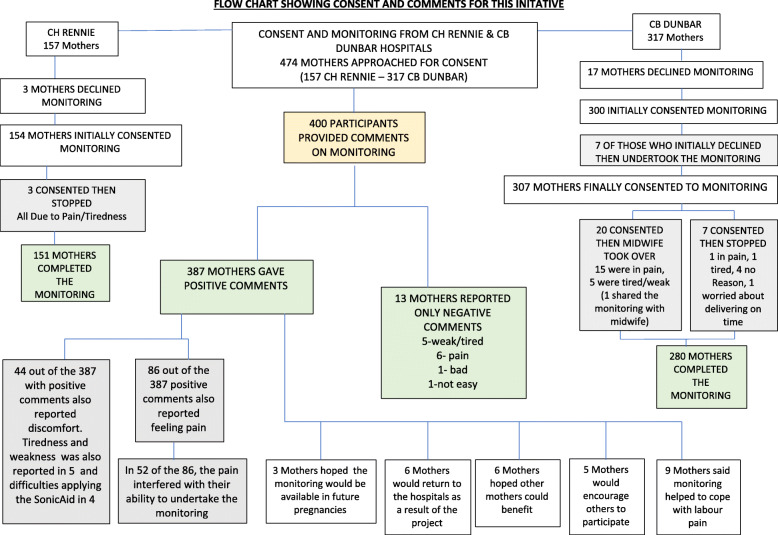
Flow chart showing consent and comments for this initative

#### CH Rennie hospital data

At CH Rennie Hospital, data were collected from 21 March 2018 until 24 October 2018. One hundred fifty-seven mothers were approached for consent. Three declined [one because of too much pain); for two no reasons were given. In the 3 women who declined, only fetal monitoring as part of the partograph, continued to be undertaken when staff time was available.

Three participants had consented but, after monitoring for 12, 8 and 36 contractions respectively, stopped monitoring because of pain and tiredness.

#### CB Dunbar hospital data

At CB Dunbar Hospital, data were collected from 31 July 2017 until 24 October 2018. Three hundred seventeen mothers were approached for consent. Three hundred gave their informed consent and 17 declined. In 7 of the 17 who declined, the mothers subsequently changed their minds and undertook monitoring for the rest of their labour. In one of these latter cases, the mother shared her monitoring with her midwife and obstetric clinician. Three hundred and seven mothers thus continued with monitoring their fetus and 10 declined. In 4 of these 10 cases, where the mother said her pain was too much to allow her to do it, and with the woman’s permission, the obstetric clinician and/or midwife continued to undertake the monitoring with the sonicaid. In one of these latter 4 cases a student midwife undertook all of the monitoring and identified a change in FHR (see Table 4). In the other 6 cases, no FHR monitoring was undertaken except as part of the partograph when staff time was available.

Out of the 307 mothers who continued monitoring (see Fig. 3) and in an additional 7 cases, the mothers subsequently stopped monitoring (1 through tiredness, 1 because of pain, 4 with no reasons given and 1 because she “*was worried about delivering on time”).*

In an additional 20 of the 307 cases where mothers had consented, but then stopped monitoring, a midwife or obstetric clinician took over (19 cases) until delivery or shared the monitoring with the mother (1 case). Of these 20 cases, 15 gave pain and 5 gave tiredness or weakness as the reasons.

Four hundred thirty-one of 474 (91%) mothers approached for consent were able to complete the monitoring themselves (151 from CH Rennie Hospital and 280 from CB Dunbar Hospital): see Fig. 3.

### Maternal age

Maternal age was available for 416 of 461 participants. 51 (12%) were aged under 18 years (24 mothers aged 17; 18 mothers aged 16; 5 mothers aged 15; 3 mothers aged 14; and one mother aged 13 years).

### Maternal experiences and comments (see Fig. 3)

Four hundred participants provided written or verbal (transcribed) comments on their experiences of the monitoring. Their comments included reference to the lack of any pain control, which was not available during labour in any of the public hospitals in Liberia at the time of this initiative.

Three hundred eighty-seven mothers found listening to their unborn baby a positive experience expressing one or more of the following words or phrases: *alright, not bad, good, fine, helpful, loved or liked it, happy, comfortable, gives me joy, or other positive comments such as “Thank you”*. A selection of these comments is shown in Tables 3, 4, 5 and 6, including some from mothers who identified changes in FHR. The complete set of comments from all participants are available in the supplementary information (Additional file 2).

Thirteen participants reported only negative comments: 5 reported weakness or tiredness (including feeling nauseated in one case), 6 reported pain (which in 3 interfered with the monitoring), 1 said it was not easy and 1 said it was bad.

*Selected maternal comments from mothers at both Hospitals (additional maternal comments following changes in fetal heart rate are reported in* Tables 4 and 6*below).*

Out of the 387 providing positive comments, 86 also reported how much they were affected by pain or severe pain. This pain interfered with their ability to undertake the monitoring in 52 of the 86 (60%).

Within the 387 with positive comments, 44 women also reported discomfort, 5 reported tiredness and weakness and 4 reported difficulties applying the sonicaid.

Three mothers said that they would hope that monitoring would be available during their future pregnancies. Six mothers said they would return to the hospitals to deliver in the future as a result of the project. An additional 6 mothers said they would hope that monitoring will continue to be available so that other mothers can benefit and 5 said they would encourage other mothers to participate. Nine participants said monitoring helped them to cope with labour pain.

###  Technical and administrative problems identified

As the forms were to be completed by busy obstetric clinicians, not dedicated researchers, forms were designed to record the most relevant information for the purposes of the study objectives rather than detailed information which, although useful, was not necessary for the purposes of this initiative and could have taken the health workers away from their clinical work.

The initial design of the tick sheet documenting each contraction monitored did not always allow enough space to record every contraction and obtaining extension sheets was sometimes a logistic problem. To minimise the workload of the scarce midwifery workforce, forms were also re-designed to record clinical data that were appropriate but not excessive given time restraints.

Due to a communication problem, 57 mothers at CB Dunbar Hospital (between 19 Feb 2018 and 8 May 2018) incorrectly monitored their FHR every 30 min (similar to the partograph). However, unlike the partograph, monitoring was always undertaken for approximately 60 s immediately following the nearest contraction to each 30-min window. We did not identify any clinical differences or differences in experiences between the 57 women who monitored the fetal heart every 30 min and the women who monitored after every contraction.

### Birth/delivery data

In 474 mothers approached for consent, there were 33 caesarean sections (7.0 %) including 14 with FHR changes. There were 20 vacuum deliveries (4.2 %) including 8 with FHR changes, (the latter included a mother with a stillborn baby identified during the training in the use of the sonicaid, confirmed by ultrasound scan).

*Clinical information and outcomes where FHR changes were identified by monitoring* Table 4.

Fetal death was identified on admission during training in the use of the sonicaid in one mother (see Table 4 below).

**Table 4:** Clinical information and outcomes of FHR changes identified by monitoring. Abbreviations: see list

Maternal age group (years)	Parity	Change in FHR identified	Action taken	Apgar scores at 1 and 5 min	Resuscitation given	Maternal comment
29–39	G5P2	By mother. FHR 115 with meconium Confirmed by MW	Lateral tilt and intravenous cannula with NS bolus Vacuum delivery	9 and 10	No	According to patient she lost her fetus during past pregnancy. Here she was happy when she noticed her fetal heart beat was dropping and the quick response that was processed
29–39	G3P2	By MW and mother during training in the use of the sonicaid at time of admission. No FHR was identified and there was 3+ meconium	Ultrasound confirmed IUFD. Vacuum delivery was undertaken	NA	NA	NA
17 and below	G2P0	By mother at 46th contraction FHR 109 with meconium	Cervix fully dilated and urged to push. NVD occurred.	4 and 7	Yes. Bag and mask ventilation, adrenaline and chest compressions for 10 min. Admitted to the NNU for post resus care and close monitoring Developed convulsions due to HIE and treated successfully with phenobarbital and recovered and was feeding normally at discharge home aged 7 days.	Listening to my baby heart was good. It help me to know that something was happening to her. No problem with it. Thank you.
18–28	G2P0	By mother FHR 119 at 49th contraction. There was + meconium present	Vacuum delivery	7 and 10	No	I like the thing I was doing but it was hard to do because of the pain.
18–28	G2P1	By mother FHR 119, 117, 116. No meconium. Patient was not progressing at this stage. 2 cm cervical dilatation with mild contractions.	MW/OC took over the monitoring due to the bradycardia. Doctor contacted. Patient was laterally tilted, given oxygen, D50%, hydrated and rushed to the OR for CS.	7 and 10	No	Thank you for this program. If not so my baby was going to die. The only thing that the pain.
18–28	G2P1 No previous CS	By mother FHR 163–165 with meconium. Signs of Bandl’s ring and obstructed labour with haematuria identified.	Not receiving oxytocin. Emergency CS	9 and 10	No	Thank you for saving my life and my baby. It really helpful to listen to my baby heart to know what was happening to me.
18–28	G3P2	By mother FHR 119,110,118. No meconium. OC and doctor contacted and confirmed bradycardia	Given facial oxygen, lateral tilt, N/S and D50%. Patient was 6 cm dilated at this stage. Emergency CS	8 and 10	No	I feel good when I was listening to my baby heart. It help me to know what happen to my baby.
18–28	G1P0	By mother at 46th contraction FHR 117, then 114, then 116, then 113. No meconium. Fully dilated but descent only minus 2	Lateral tilt, D50%, oxygen, NS and FHR still below 120 She sat on birthing chair for 10 min and when head reached below 0 station (re: ischial spines) vacuum delivery was successfully undertaken	7 and 10	No	Thank you for what you bringing because when it was not because of it I was not coming to know say my baby heart was not beating good. That just the pain was giving me hard time thank all.
17 and below	G1P0	FHR found to be 95–100 by mother, FHR was repeated by midwife and confirmed low, 95–98, and Doctor on call was also informed.	Patient was placed in a left lateral tilt position Patient was reviewed and decision to CS was taken for fetal distress plus prolonged labour	6 and 9	None	Not requested at this stage in programme
18–28	G3P1	Mother reported a change in FHR but when checked by MW found FHR to be normal at 142. Meconium was present	Doctor informed but no action was considered necessary	6 and 10	None	Not requested at this early stage in programme
17 and below	G1P0	On 11th contraction mother reported slow heart rate. MW was contacted but she found FHR was 153. There was no meconium the OC was contacted.	Mother’s membranes were ruptured and vacuum delivery undertaken	7 and 10	None	Not requested at this early stage in programme
17 and below	G1P0	Mother noted change in FHR and contacted MW on 15th contraction. MW noted FHR 118 and informed OC. Meconium was present repeat fetal heart rate was 105.	Mother put in lateral tilt position and informed Dr. who reviewed patient and found fetal heart rates 110, 105, and 108. Emergency CS was performed	8 and 10	None	Not requested at this early stage in programme
18–28	G2P1	On 11th contraction mother noticed bradycardia. Midwife confirmed FHR 118 Grade 3 meconium was present. Patient placed in left lateral position and called OC. OC found FHR to be 110.	Left lateral tilt. Cervix was fully dilated and vacuum delivery was undertaken.	6 and 9	Bag and mask ventilation. Admitted NNU for 5 days and treated for sepsis.	Not requested at this early stage in programme
18–28	G1P0	Yes - by MW following being declined by mother FHR 95–100 on two successive occasions	Lateral tilt and subsequent CS for non-reassuring FHR	5 and 7	Bag and mask ventilation and admitted to NNU. No HIE and went home.	Following initial consent, patient later declined to monitor her FHR. Says she was tired of monitoring.
18–28	G3P2	Mother on 14th contraction noticed change in FHR to 102. And complained of weakness. She called for help and FHR was102. No meconium was present.	OC contacted, lateral tilt and intravenous (IV) cannula with 500 ml of Ringer Lactate given. Normal vaginal delivery followed.	6 and 10	This baby was resuscitated for 5 min with bag and mask ventilation and then transferred to the NNU where he was immediately placed on nasal CPAP and an IV line was opened to serve antibiotics because amniotic fluid was also purulent and foul smelling. IV fluid (Dextrose 10%) was set up. Baby was managed for 7 days in the NNU and was discharged home with good outcome.	According to mum monitoring is hard at certain times. She knew her baby's heart rate was low and we took quick action and now the baby is in her hands so she thank the organisation.
17 and below	G1P0	On the 14th contraction the mother called the MW because the FHR was low. The MW confirmed FHR 98, called for help and undertook lateral tilt. Meconium was present.	The OC was contacted. She opened IV line and gave R/L 1000 mL, informed the doctor on call. The doctor came and assessed the patient and said we should prepare patient for CS. CS was done for prolonged labour and abnormal FHR.	5 and 10	Neonate was resuscitated for 7 min by bag and mask ventilation before transferring to the NNU. She was placed on nasal CPAP for 24 h and was also managed for risk of sepsis. Neonate improved after 8 days and was discharged.	According to mum it is okay because this help the doctor nurses to take quick action
17 and below	G1P0	On the 7th contraction, mother detected fetal bradycardia 105 bpm. MW called and checked and confirmed FHR 105. Meconium was present. Grade 3 OC was called.	Lateral tilt was undertaken and fast vaginal delivery arranged as 9 cm cervix dilated. Birth weight 1.9Kg small for dates.	7 and 10	Baby was resuscitated for 2 min by bag and mask ventilation and then transferred to NNU. She was placed on nasal CPAP for 24 h and patient condition improved. Baby was also managed for risk of neonatal sepsis because mother’s amniotic fluid was purulent, foul-smelling during delivery. The baby was discharged home after 10 days with a weight of 2.3 kg	Patient initially declined procedure but later on she was encouraged to do it herself and everything went well
18–28	G5P4	On 6th contraction, mother detected bradycardia 108 bpm. MW confirmed FHR 108. Meconium was present.	OC contacted. Lateral tilt performed. IV cannula inserted and given NS 500 ml. Normal vaginal delivery occurred.	5 and 8 Male	Bag and mask ventilation given. No HIE occurred but he needed 5 days of antibiotics for umbilical infection.	Patient worry when the heart rate was reducing but at last she was happy because her baby came through
29–39	G5P3	On 2nd contraction monitored mother identified rapid heart rate. MW confirmed FHR 190 and called for help,	Doctor called and attended. Lateral tilt and IV cannula and N/S 500 ml set up. Vacuum delivery was undertaken.	6 and 8	Neonatal clinician was called and baby resuscitated with bag and mask ventilation and recovered within 1 min. Responded well and taken to NNU for suspicion of sepsis. No HIE.	Mother said she was happy with the monitoring because she could have had a dead baby if she didn’t monitor. She’s also asking other mothers to accept and be part of the process
17 and below	G2P1	On 6th contraction, Mother reported fall in HR. MW confirmed FHR 109 Meconium present.	Lateral tilt applied and IV cannula inserted with R/L 500 mls plus Dextrose 50% 30 ml. OC contacted and quickly delivered the baby vaginally.	6 and 7	Mildly depressed but no resuscitation needed. Neonatal clinician continued monitoring and care.	Patient was very happy because she call for help and action was taken quickly by the OB clinician and her baby was save.
18–28	G3P0	On 27th contraction, Mother detected slowing of FHR. MW confirmed FHR 109. Grade 2 meconium was present. Dr. on call contacted.	Lateral tilt and IV cannula inserted. R/L 500 mls given IV. Doctor arrived and undertook CS.	7 and 10	Resuscitated for 2 min with bag and mask ventilation.	According to mother she was very happy, and she told everybody thanks because of the monitoring her baby was saved
17 and below	G1P0	On 7th contraction mother noted fast heart rate. MW confirmed FHR 167. Patient came in fully dilated but evidence of obstructed labour due to persistent occipito-posterior malposition.	Lateral tilt and IV cannula inserted. NS 500 mls given IV. Doctor arrived and undertook CS.	9 and 10	None needed	Mother was happy to hear her baby heart beat because she stay in labour for long and worry about her unborn baby
40 and above	G9P8	On the 7th contraction mother with MW noted a slow heart rate FHR 102. Meconium was present and a cord prolapse identified.	The OC was notified and implemented knee chest position and inserted NS 300mls into the bladder to reduce cord compression. IV cannula was inserted and NS 500 mls given. A CS was then undertaken.	6 and 10 Depressed breathing.	Resuscitated for 1–3 min with bag and mask ventilation. Taken to NNU as 1.7 Kg and 30 weeks’ gestation No HIE. Home after 14 days	According to mother monitoring is good but she cannot continue it herself due to pain. At last she said it help her with a live neonate
17 and below	G2P1 previous CS	On 12th contraction, Mother reported slowing and with MW reported a FHR 124. Meconium present Grade 3 Then FHR dropped to 119 bpm	OC was called and after lateral tilt established IV line and gave 500 ml NS. A CS was then undertaken.	7 and 8	No resuscitation needed but foul-smelling amniotic fluid at CS led to NNU admission and IV antibiotics.	Mother agreed to the process, she started it but discontinue due to pain and was helped by midwife and OB clinician. Mother said it’s a good thing, it help her have a live baby
29–39	G1P0	Induced for post date. On the 8th contraction mother noted a slow heart rate. MW contacted and confirmed FHR 110. Meconium was present. OC informed and FHR was 112. Cervix fully dilated.	Lateral tilt and placed in delivery room for vacuum delivery. However, within 5 min delivered NVD spontaneously. A very short umbilical cord was present.	5 and 7 Depressed breathing	Resuscitated for 5 mins with bag and mask ventilation and taken to NNU and given antibiotics. Later became stable and discharged.	The monitoring was good, it is a good idea and I hope it will continue because it will save a lot of babies as it did mine. Sometimes the midwives are busy so this will help them and help us the mothers too. Mother was hospital medical director ‘s sister in-law
29–39	G5P4	On the 30th contraction mother noted a slow heart rate. MW confirmed FHR 118.	MW performed lateral tilt and informed the OC and set up IV infusion of R/L 500 ml. Dr. ordered repeat and FHR 106. Cervix only 4 cm dilated. Descent 3 / 5. Discussion for CS was done but no CS materials available, so patient was referred to another hospital.	8 and 9	None needed after CS at referral hospital	I like listening to my baby heart but I don’t know if my baby will live again now that I am going to a different hospital. Outcome at second hospital after CS was good for mother and baby.
18–28	G1P0	On the 20th contraction OC and student MW confirmed a slow FHR 105. No meconium seen.	Lateral tilt was undertaken. The cervix was already 10 cm dilated and there were poor maternal efforts. An IV cannula was inserted and she was given 30 ml dextrose 50%. Baby was delivered by vacuum.	5 and 6	Yes, by neonatal clinician bag and mask ventilation for 5–10 min. Admitted to NNU for neonatal depression. Neonate recovered quickly on nasal CPAP. Improved and went home well.	Mother had declined monitoring but this was done by student MW.
18–28	G1P0	On 30th contraction mother noted slowing of FHR. There was no meconium at this time. MW and OC identified FHR of 115, 118,122.	Lateral tilt and Doctor notified. An IV cannula inserted and given N saline 500 ml plus Dextrose 50% 30 ml. The cervix was 10 cm dilated. OC did vacuum with Dr. present but failed 3 times. Dr. and OC proceeded to immediate CS. Intraoperative meconium was present	5 and 7	Bag and mask ventilation for mild respiratory depression. Recovered rapidly and went home.	The monitoring is good but I was not able to do it all by myself because of the pain and my foot pain. Yes my baby is living so it help. No problem with it but the pain can be too much.
18–28	G4P0	On 51st contraction mother noted slowing of fetal heart rates. MW recorded FHR 109, 178,120,110,181,102,130 Meconium was present	Lateral tilt was performed, and OC notified. IV fluids were started, and 30 ml of 50% dextrose given IV. The doctor was also called and due to FHR changes, high station on vaginal examination, and bad obstetric history (G4P0) proceeded with the OC to CS.	8 and 10	No	The monitor help me to inform the midwife that my baby was not breathing good. So I see it to be good for all the big belly with stomach hurting pain.

Abbreviations are defined in the list given earlier in the manuscript

Table 4 describes the clinical information and outcomes relating to identified FHR changes. Changes in FHR were reported in 28 of 461 participants (6.1%,) which in two cases were not confirmed by the attending midwife, giving 26 confirmed cases (5.6%). Twenty-four of these FHR changes were first identified by their mothers. In 23 of the 26 confirmed cases, the FHR decreased and in 3 the FHR increased. Two changes related to unrecognized obstetric complications, with one mother found to have Bandl’s ring with obstructed labour and the other, cord prolapse.

All 26 neonates with changes identified in FHR survived, including 13 requiring resuscitation at birth. Twelve neonates were admitted to the neonatal unit. One neonate developed birth asphyxia/HIE but the immediate clinical outcomes of the 25 other neonates were good and all 26 were discharged home apparently well.

One of the 26 changes in FHR was identified by a midwife who had taken-over monitoring from a mother who became too tired to continue. In a second case, the mother had declined to undertake monitoring herself but had consented to the monitoring being undertaken by a student midwife.

In 16 of the 26 with confirmed FHR changes, plus 1 intrauterine fetal death detected on admission, there was accompanying meconium-stained liquor.

Thirteen of the 26 (50%) neonates with prior FHR changes had low Apgar scores and needed resuscitation. There were no deaths following resuscitation. One baby had convulsions managed with Phenobarbital, recovered, and was feeding normally at discharge home aged 7 days. None of the other 26 neonates developed birth asphyxia (also known as Hypoxic Ischaemic encephalopathy-HIE). However, long-term infant follow-up was not undertaken.

Seven of the 26 neonates with confirmed FHR changes, plus 1 who had suffered intrauterine fetal death, were born by vacuum, 14 by Caesarean Section (CS) and 5 by vaginal delivery. In one case, CS followed a failed vacuum delivery.

*Clinical information and outcomes in 3 newborn infants needing resuscitation at birth where mothers had declined to participate in FHR monitoring.* Table 5.

**Table 5:** Clinical information and outcomes in newborn infants needing resuscitation at birth where mothers had declined consent to participate in the FHR monitoring. For abbreviations see list

Maternal age (years)	Parity	Change in FHR identified on partograph	Delivery	Apgar scores at 1 and 5 min; Wt. of baby	Resuscitation given	Maternal comment	Other possibly relevant information?
29–39	G3P2	None	Normal vaginal delivery	2 and 3 3.4Kg	Resuscitated with bag and mask ventilation, chest compressions and oxygen Admitted NNU but later died aged 2 days from HIE	None	Mother declined monitoring and staff could not take over
18–28	G2P1	None	Normal vaginal delivery	4 and 6 Depressed 2.8Kg	Resuscitation was done with bag and mask ventilation and was taken to the neonatal ward. Treated with antibiotics. Outcome was good and discharged.	None	Patient declined to continue her fetal heart rate monitoring even though she did monitor the first contraction
18–28	G1P0	None	Normal vaginal delivery	2 and 6 Very depressed	Resuscitated by bag and mask ventilation by neonatal clinician Died aged 3 days from HIE	None	Patient declined to continue her fetal heart rate monitoring even though she did monitor the first contraction

Abbreviations are defined in the list given earlier in the manuscript

In one such case, the baby was born with Apgar scores of 2 at 1 min and 3 at 5 min and, despite resuscitation, died of HIE in the neonatal unit aged 2 days. In 2 other cases, there were low Apgar scores at 1 and 5 min (4 and 6; and 2 and 6) and the babies needed resuscitation. Both were admitted to the neonatal unit. One responded well to resuscitation with no evidence of HIE and was discharged home well. The other died aged 3 days from birth asphyxia/HIE.

*Clinical information and outcomes in newborn infants needing resuscitation at birth where monitoring had not identified any FHR changes.* Table 6.

**Table 6:** Clinical information and outcomes in newborn infants needing resuscitation at birth where monitoring had not identified any FHR changes

Maternal age (years)	Parity	Change in FHR identified	Delivery	Apgar scores at 1 and 5 min; Wt. of baby	Resuscitation given	Maternal comment
18–28	G2P1	None. Monitored only every 30 min immediately following 14 contractions	Preterm labour and normal vaginal delivery	5 and 7 depressed at birth 1.8 Kg	Neonatal clinician called, resuscitated with bag and mask for 12 min and taken to neonatal ward No HIE	Mother said the monitoring help her with her baby, she got a live baby. She was willing and cooperative and ask other mothers to accept the monitoring
18–28	G1P0	None. Monitored only every 30 min immediately following 13 contractions	Normal vaginal delivery	5 and 10 depressed at birth 3.9Kg	Neonatal clinician was called, did 10–15 min bag and mask ventilation. Oxygen saturation 54%. Admitted NNU. No HIE and went home aged 7 days	According to mother the monitoring is good, it help her deliver her baby live. She was interested in doing it
18–28	G2P1	None. Monitored only every 30 min immediately following 11 contractions	Normal vaginal delivery	7 and 10 2.8 Kg	Bag and mask ventilation used for 5 min and then recovered. No HIE	Appreciated the listening to her baby until birth. She recommended that all labouring mothers should be able to listen to their fetus during labour
29–39	G2P1	No abnormality detected following 12 contractions	Vacuum delivery unable to push	5 and 8 3.9Kg	Resuscitated by bag and mask for 10 min. Admitted NNU and given 7 days antibiotics. No HIE.	It help because with all the pain I refused to listen to them. I still got my baby by talking to me good. I found it very good because it help me in getting my baby. No problem.
18–28	G2P1	No abnormality detected following 15 contractions	Normal vaginal delivery	2 and 0 1.3 Kg 34 weeks’ gestation	Resuscitated by bag and mask ventilation plus chest compressions for 25 min. But then died.	I like the monitoring. I enjoy listening to my baby even though he didn’t survive
17 and below	G1P0	No abnormality detected following 58 contractions	Normal vaginal delivery episiotomy for baby stuck at perineum	5 and 7 2.5Kg	Resuscitated by bag and mask ventilation by neonatal clinician for 8 min then improved and discharged. No HIE.	I see the monitoring good for me and my baby because it my make me to know that my baby is still living. 9th grade student
18–28	G2P1	No abnormality detected following 42 contractions	Vacuum delivery for poor maternal effort	6 and 8 3.1 Kg	Bag and mask resuscitation for 7 min. Baby was admitted to the NNU for observation. No HIE but had malaria and was treated for 10 days and then discharged well.	*OC was contacted during labour. OC/MW took over monitoring: patient says she can’t continue due to the pain. The contractions is too frequent and strong.* I like the monitoring. It make me born a living baby but it is hard to do. It is hard to be in pain and holding the machine.
18–28	G1P0	No abnormality detected following 12 contractions	Vacuum for exhaustion: couldn’t push	7 and 8 3.3 Kg	Bag and mask resuscitation one-two breaths only before baby breathed. Not admitted to NNU.	I find it good. It help me because my baby is alive. No problem with it.

Abbreviations are defined in the list given earlier in the manuscript

In addition to the neonates requiring resuscitation where FHR changes had been detected, 8 other neonates *without detected changes* in the FHR required resuscitation (Table 6). Two were of low-birth weight (1.8 and 1.3 Kg). Despite resuscitation for 25 min one of the low birth weight babies died at birth. None of the 7 babies who survived showed evidence of birth asphyxia/HIE.

In one mother, a vacuum delivery was undertaken for inability to push during the second stage of labour., Apgar scores were 5 and 8 and the baby required  10 min of bag and mask ventilation. He was discharged home aged 7 days well. No evidence of birth asphyxia/HIE was evident on clinical assessment.

Three cases were born following vacuum delivery and 5 cases by vaginal delivery. In one mother progress of her fetus was arrested at the perineum and an episiotomy was undertaken to expedite vaginal delivery.

Three of these 8 neonates had been monitored in utero only every 30 min in a temporary deviation to the protocol because of a communication problem with one of the trainee obstetric clinicians but none developed birth asphyxia/HIE.

### Costs associated with collecting data

The costs of the project were low. The fetal doppler monitors (Sonicaids: 12 in total) cost USD 40 each. Rechargeable AA batteries were used. Additional costs included paper and printing for the consent, data collection and monitoring forms including the internet costs of scanning and sending them to MCAI for analysis, and KY jelly (or locally available clear hair gel) for interfacing the ultrasound probe with the abdomen: commercial ultrasound gel was too expensive.

### Missing data

Because of problems with the completion of medical records and the work pressure on the health workers involved, it was sometimes difficult to fill the gaps of any missing information, such as birth weights, retrospectively. Every effort was made by the management committee to minimise missing data, especially regarding maternal and neonatal outcomes.

## Discussion

### Summary

This initiative aimed to assess whether mothers were able to undertake fetal heart rate monitoring of their unborn babies immediately following the end of every contraction during labour in two rural public hospitals in Liberia, a country with extremely poor resources (both human and material). As 93% of consenting mothers were able to undertake the monitoring themselves until their baby was born, the results suggest that this approach is feasible.

The comments and feedback from the participating women and adolescent girls (not just those selected in the Maternal Comment sections of the tables but also the complete set of comments provided in Additional File 2 as supplementary information), showed that the overwhelming response (387 out of 400) was positive, with several comments reflecting how the monitoring helped a women/ adolescent girl “feel strong”, cope with the pain of labour, or made her feel closer to her baby.

In our experience, it is most unusual for mothers in Liberia (as in many other low-resource settings where the hospital workforce is so limited and stretched) to be asked for their opinions on their experiences of labour and how it was managed. We asked for their opinions in an open way and did not raise the question as to whether they would prefer any alternative approaches to fetal monitoring, as stretched resources, lack of health workers, and subsequent high workloads, made such alternative approaches unfeasible. Also, as explained in our Background, without this approach, many women would not have had her fetus routinely monitored because of the scarcity and high workload of midwives.

To go through a pregnancy only to have a fetus or baby die because of a preventable condition that might have been detected by adequate fetal monitoring is a tragic and traumatic experience. This initiative may have helped to prevent some deaths, while helping women/adolescent girls feel involved in what was happening to them and their babies during labour.

### Interpretation

Studies of mothers assisting midwives in monitoring the FHRs of their unborn babies have not been previously reported, as far as we can identify, so it is difficult to compare our results with other studies.

This initiative confirmed that midwives responded to alerts from mothers regarding changes in FHR in a timely fashion and that trained, senior, health professionals (obstetric clinicians and doctors) were able to intervene appropriately and promptly. In 26 out of 461 women undertaking monitoring (in one undertaken with consent by a student midwife and the other by the midwife who took over from the mother who became tired), confirmed changes in fetal heart rates were identified. Actions to improve the placental circulation (such as lateral tilt, oxygen and intravenous fluids), and, where possible, expedited delivery by vacuum or Caesarean section were undertaken following standard obstetric management as recommended by WHO [21].

All 26 neonates with changes in FHR survived, including 13 requiring resuscitation at birth. Ten neonates were admitted to the neonatal unit. One neonate developed birth asphyxia/HIE, but the immediate clinical outcomes of the 25 other neonates were good and all 26 were discharged home apparently well. However, long-term infant follow-up was not undertaken and should be in the future. The presence of neonatal clinicians and a functioning neonatal unit at CB Dunbar hospital were particularly valuable. Neonatal units that can provide advanced neonatal care by appropriately trained nursing staff (also a form of task-sharing) have now been established at 2 other rural county hospitals in Liberia.

In two mothers, the identification of FHR changes revealed previously unrecognized life-threatening obstetric complications. More experience may identify whether maternal FHR monitoring can consistently identify obstetric complications. Our results support Hofmeyr and colleagues [16] who stressed the importance of task-sharing in the integration of obstetric and neonatal care. Rapid access to effective obstetric management if FHR changes are identified must be ensured. Fetal monitoring in isolation has little value without an available health support system so that appropriate clinical intervention can be promptly undertaken. This situation may not be the case in some resource-limited settings, limiting the applicability of maternal fetal monitoring.

### Limitations

Comparing figures with those from Tanzania [14] and from Sub-Saharan African countries [1, 12, 13, 15], the absence of intrapartum stillbirths in the 461 fetuses monitored is encouraging, as is the low prevalence of birth asphyxia/HIE. However, given no comparator, we cannot conclude that stillbirths and birth asphyxia/HIE can be prevented or reduced by maternal FHR monitoring. However, in reality, the lack of comparator data is represented by almost no fetal monitoring in public health hospitals in Liberia. We considered having a control group for this study, but decided that given the situation described in the “Background”, such an approach would have been difficult. However, partway through this initiative, data on neonatal deaths and intrapartum stillbirths began to be collected by the Ministry of Health in Liberia. In the future, such data may provide control information for those maternity facilities where maternal FHR monitoring has not yet been undertaken.

Since we do not have robust baseline (comparator) data we cannot at this stage comment on whether Caesarean Section or operative vaginal delivery rates were increased or decreased by maternal FHR monitoring.

This initiative was conducted in a “real world” setting and suggests that maternal fetal monitoring can be incorporated into the daily work of a busy hospital maternity unit. The constant presence of obstetric clinicians was of considerable assistance, but such experienced health professionals may not always be available and alternative plans to address the health-workforce-shortage may be needed. Even with this additional cadre, communication and adherence to the study protocol were sometimes difficult, depending on the enthusiasm and involvement of all health professionals. Feedback of results to the midwifery workforce appeared helpful. Although we did not explicitly seek the views of attending midwives, which was a major limitation, the finding that midwives sometimes took over the monitoring if a woman was too tired or in too much pain to do it herself, shows their engagement in the process. More information from midwives could also be useful in helping to improve this technique, make it more sustainable, and give a further guide to the additional time needed to undertake maternal-fetal monitoring.

The potential for blaming mothers if there is an adverse outcome for their baby due to perceived failings in their self-monitoring is a very important consideration. However, there was no evidence that this situation happened, and, in our view, potential blame is unlikely as sadly, neonatal death and stillbirths are such common experiences in Liberia, that such events are almost expected.

We appreciate the frequency of monitoring can be onerous for some women, but there was no provision for birth partners or other relatives to be with women during labour, because of shortage of space and lack of privacy. We are planning to address this situation in the future.

### Developments and possible ways forward

The long-term solution to fetal monitoring and improving the care of women in labour and their babies is undoubtedly to increase the availability of trained midwives. However, as explained in the “Background” to this paper, and especially the Global WHO/World Bank data concerning midwives and nurses [2], given the current situation in Liberia and many other low income settings with minimal midwives and nurses, a vast amount of resources and an increase is suitably trained health workers would be necessary to meet this goal, which is not possible in the short-term. However, once and if this situation is achieved, given the positive responses of participating mothers, we would continue to consider that the involvement of mothers in fetal monitoring may work well in collaboration with the enhanced role of skilled birth attendants.

The need for resuscitation in 8 of 461 (1.7%) of babies born without FHR changes identified by maternal monitoring, requires future research to ascertain whether changes are being missed or whether in some cases there are no measurable FHR changes identifiable by this monitoring technique in fetuses who subsequently require resuscitation. In particular, it is important to know whether mothers and/or midwives can monitor reliably after every contraction with the same quality during the end of the first and second stages of labour when contractions can be much more painful.

When appropriately undertaken and documented, with timely responses by care givers, the partograph is of major value in monitoring the progress of labour and safety of mother and her fetus. In support of Hofmeyr et al. [16] who stressed the importance of task-sharing (in this case involving mothers), our findings show that it is feasible for pregnant women to undertake fetal heart monitoring during labour so that changes in FHR in relation to the end of every contraction are monitored. A decrease in FHR that persists after the end of a contraction, or continues after a contraction, is more likely to be pathological (Type 2 decelerations). WHO recommendations in place at the beginning this initiative [20, 21] specified FHR documentation on the partograph every 30 min in the first stage and every 5 min in the second stage of labour and not with every uterine contraction, as in our initiative. Moreover, in these earlier guidelines [21], WHO had recommended that the FHR be listened to immediately following the end of a contraction ONLY during 1) the initial assessment of the mother during labour; 2) when malpresentation or malposition are present; and 3) when inducing or augmenting labour. However, during the implementation of this initiative, in February 2018, WHO updated its guidelines on intermittent FHR auscultation in labour, now recommending auscultation every 15–30 min during the first stage, and every 5 min during the second stage of labour stating that auscultation should begin during a contraction and continue for at least 30 s after the contraction has ended [7]. These latest guidelines also recommend that if the FHR is not always within the normal range (110–160 bpm), auscultation should be prolonged to cover at least 3 contractions and also recommend recording the baseline FHR and the presence or absence of accelerations and decelerations [7]. These updates in guidelines are welcome but, in our opinion, are quite complex, difficult to implement in facilities where there are few midwives, and do not concentrate on the most important time for intermittent auscultation, that is, at the end of **every** contraction. Our approach which involved auscultation from the end of every contraction, was relatively easy to teach, convenient and feasible for individual mothers to undertake, and covered the most critical time for monitoring. For low resource settings, this approach may merit consideration by WHO and the wider international community.

Future expansion of the project to include the possible role of female relatives and traditional birth attendants in supporting the mothers during monitoring, especially at times when contractions are particularly frequent and painful around the end of the first stage and during the second stages of labour, could be helpful. Unfortunately, the lack of space, privacy and resources such as toilets in the labour and delivery wards at present does not readily support this approach. Helpers such as traditional birth attendants or nurse aides may also be of value in collecting baseline/comparative data, and feedback from midwives.

An introduction to self-fetal monitoring during labour at antenatal visits would be beneficial, noting that a significant proportion of pregnant women in rural Liberia do not always attend their assigned antenatal clinics.

The mothers’ comments on the lack of any pain control were of great concern and several mothers were clear about the need for pain control during labour. We have now followed up on the need for adequate pain control during labour within the Liberian Ministry of Health and Social Welfare and have started a clinical audit on the use of intravenous paracetamol at CB Dunbar Hospital.

Some mothers stated that they would encourage relatives and friends to attend the hospitals involved in this initiative so that they might benefit from the fetal monitoring process. Given the encouraging findings of this study, we suggest that other hospitals in low resource settings, where there are few midwives and consequently, inconsistent fetal monitoring on the partograph, consider introducing maternal FHR monitoring, possibly assisted by relatives where circumstances allow. Although a small minority of mothers expressed negative comments, there does not appear, to the best of our knowledge, to have been have any negative clinical consequences to maternal fetal monitoring. Given the circumstances in countries where there are so few nurses and midwives, this approach supports realizing the rights of adolescent girls and women by enabling them to become more involved in their health care and the welfare of themselves and their unborn babies.

This initiative is continuing in both CH Rennie and CB Dunbar hospitals with further expansion planned in the near future to three additional rural maternity hospitals in Liberia.

## Conclusions

The most promising findings of this initiative were the ability of the mothers to detect fetal heart rate changes and for midwives, obstetric clinicians and doctors to urgently respond to confirmed changes with appropriate clinical actions. The absence of intrapartum stillbirths, and the low rates of resuscitation and subsequent birth asphyxia/HIE in neonates who had been monitored are encouraging. A welcome finding was the positive comments made by 387 of 400 mothers about their involvement in the monitoring of their own unborn babies. If further studies in other settings, involving much larger numbers, continue to show these benefits, this new approach may help to reduce the devastating problems of intrapartum stillbirth and birth asphyxia / HIE that are so prevalent in low resource settings.

## Supplementary information

**Additional file 1.** Master copy of the consent form, fetal heart rate monitoring and data collection and maternal comment form. This form describes the way in which data were documented and collected on each participant. The consent form provides the information that was either read by each participating mother, if literate, or read to the mother by the obstetric clinician. The form could either be signed by the mother or she could provide her fingerprint from an ink pad. The FHR monitoring form was given to each mother who was asked to tick each time she listened to the FHR for approximately one minute following the end of every uterine contraction. If the mother considered that the FHR had changed, she would notify the midwife caring for her who would check the FHR, count it, and write down her own findings on this chart. If the midwife considered that there was a potentially clinically relevant change in FHR she/he immediately notified the on-call obstetric clinician or doctor. The data collection form recorded a summary of potentially relevant clinical data on the labour, delivery, neonatal Apgar Scores at 1 and 5 min, and any resuscitation given to the baby. Recorded on this form was any admission of the neonate to the neonatal unit and any treatment given to the baby. The form also includes a section in which the comments made by mothers on their experience of the monitoring were recorded either by themselves or as transcribed by the attending midwife or obstetric clinician.

**Additional file 2.** Table of all maternal comments on their experience of undertaking monitoring of their unborn babies during labour categorised by age groups. This file describes the actual comments made on the monitoring process by each mother, either written directly or transcribed for illiterate mothers by the attending obstetric clinician.

## Data Availability

The datasets used and/or analyzed during the current study are available from the corresponding author on reasonable request.

## Abbreviations

CBD: CB Dunbar Hospital
CHR: CH Rennie Hospital
CPAP: Continuous Positive Airways Pressure
CS: Caesarean Section
D50%: Dextrose 50% intravenous solution
FHR: Fetal Heart Rate
HIE: Hypoxic Ischaemic Encephalopathy
IUFD: Intra Uterine Fetal Death
IV: Intra-Venous
LBNM: Liberian Board for Nursing and Midwifery
MCAI: Maternal and Childhealth Advocacy International
MOHSW: Ministry of Health and Social Welfare
MW: Midwife
NA: Not appropriate, or not available, or not recorded
NNU: Neonatal Unit
NREB: National Research Ethics Board
NS: 0.9% saline intravenous solution
NVD: Normal Vaginal Delivery
OC: Obstetric Clinician
OR: Operating Room
R/L: Ringer Lactate intravenous solution
UNFPA: United Nations Population Fund
UNICEF: United Nations International Children’s Emergency Fund
WHO: World Health Organization
