# Supplementary material for: Monitoring intrapartum fetal heart rates by mothers in labour in two public hospitals: an initiative to improve maternal and neonatal healthcare in Liberia
Source: BMC Pregnancy Childbirth. 2020 Jun 15;20:362. doi: 10.1186/s12884-020-02921-z (PMC7294611; doi:10.1186/s12884-020-02921-z)
Supplement: Supplementary file 1 — Additional file 1. Master copy of the consent form, fetal heart rate monitoring and data collection and maternal comment form. This form describes the way in which data were documented and collected on each participant. The consent form provides the information that was either read by each participating mother, if literate, or read to the mother by the obstetric clinician. The form could either be signed by the mother or she could provide her fingerprint from an ink pad. The FHR monitoring form was given to each mother who was asked to tick each time she listened to the FHR for approximately one minute following the end of every uterine contraction. If the mother considered that the FHR had changed, she would notify the midwife caring for her who would check the FHR, count it, and write down her own findings on this chart. If the midwife considered that there was a potentially clinically relevant change in FHR she/he immediately notified the on-call obstetric clinician or doctor. The data collection form recorded a summary of potentially relevant clinical data on the labour, delivery, neonatal Apgar Scores at 1 and 5 min, and any resuscitation given to the baby. Recorded on this form was any admission of the neonate to the neonatal unit and any treatment given to the baby. The form also includes a section in which the comments made by mothers on their experience of the monitoring were recorded either by themselves or as transcribed by the attending midwife or obstetric clinician. [file 12884_2020_2921_MOESM1_ESM.pdf]

Additional File part 1

MONITORING THE HEART BEAT IN ORDER TO CHECK ON THE WELL-BEING OF YOUR UNBORN BABY

CONSENT FORM

We are trying to find out whether it is practical to train mothers in monitoring the heartbeat of their unborn baby.

With your permission, we want to train you in the use of a harmless, battery-operated sensor to check your baby's heartbeat during labour for 1 minute immediately following every contraction.

There is a simple form that you can mark every time you listen to your baby's heart beat to show if it is normal or if you think it has changed.

If you detect what you think might be a changing of your baby's heartbeat, please call the midwife who is looking after you and she will check it for you in more detail.

If your midwife considers it to be abnormal, she will contact a senior staff member who will advise on whether any action is needed.

Undertaking this monitoring is entirely voluntary and if you decide either not to go ahead or if during your labour you want to stop monitoring there is absolutely no problem and your future care will continue as normal. If you refuse to undertake the monitoring, you and your baby will still be provided with the standard of care normally available during your labour.

Very many thanks for reading/listening to this explanation.

Please sign below if you agree to begin the monitoring and our specially trained midwife will show you what to do.

NAME: ………………………………………………SIGNATURE OR thumbprint…………………………………

DATE: …………………………………….

I CONFIRM THAT I HAVE EXPLAINED THE PROCEDURE AND WHEN APPROPRIATE THE MOTHER HAS READ THIS DOCUMENT.

NAME ……………………………………………………… SIGNATURE………………………………………………

Additional File Part 2 The Fetal Heart Rate monitoring data form

Date _______________ Mother's Age _____________

Time monitoring started _______________ Patient's name______________________

**Fetal heart rate by mother**		**Fetal heart rate by midwife (bpm)**	**Meconium YES or NO**	**DESCRIBE ANY ACTION TAKEN AND WHO DID WHAT**
**Normal**	**Changed X**			

Four to 6 additional similar pages added here

Additional File Part 3

Fetal monitoring project birth summary data and maternal comments

Woman's Name __________________________

Was the obstetric clinician or a doctor contacted during the labour? Yes/ No
If yes, what action was taken?

Type of birth: Normal vaginal / Caesarean / Vacuum
If Caesarean or vacuum what was the reason?

Did the midwife OR obstetric clinicians take over the monitoring of the FH from the mother at any stage? Yes/No
If yes, what was the reason?

BABY OUTCOMES

Survival: Live birth / still birth Gender Male / Female

Apgar score at 1-minute ______ Apgar score at 5 minutes ______

Was baby resuscitated? Yes/No
If **yes**, please give details

Was baby admitted to neonatal ward? Yes/No
If **yes**, what was the reason?

PLEASE ASK THE MOTHER THE FOLLOWING QUESTIONS USING THESE EXACT WORDS:
'HOW DID YOU FIND THE MONITORING? DID IT HELP WITH YOUR LABOUR? IF SO, IN WHAT WAYS? DID IT CAUSE YOU ANY PROBLEMS? IF SO, IN WHAT WAYS?
PLEASE ALLOW THE MOTHER TO WRITE HER ANSWER BELOW OR WRITE DOWN HER ANSWER **USING HER OWN WORDS**.
………
………
………
………
