# Supplementary material for: Monitoring intrapartum fetal heart rates by mothers in labour in two public hospitals: an initiative to improve maternal and neonatal healthcare in Liberia
Source: BMC Pregnancy Childbirth. 2020 Jun 15;20:362. doi: 10.1186/s12884-020-02921-z (PMC7294611; doi:10.1186/s12884-020-02921-z)
Supplement: Supplementary file 2 — Additional file 2. Table of all maternal comments on their experience of undertaking monitoring of their unborn babies during labour categorised by age groups. This file describes the actual comments made on the monitoring process by each mother, either written directly or transcribed for illiterate mothers by the attending obstetric clinician. [file 12884_2020_2921_MOESM2_ESM.pdf]

Additional File 2. Table of all maternal comments on their experience of undertaking monitoring of their unborn babies during labour categorised by age groups.

| **Age Group years** | **Comments from mothers** |
|---|---|
| N/A | The monitoring was fine, it gave me courage to go through my pain knowing my baby was fine |
| 18-28 | The monitoring was fine, it was good listening to my own baby |
| N/A | The monitoring was good I did not know I could do what the health workers (midwives) were doing. It helps me go through my pains |
| 18-28 | The monitoring was good especially from the start I felt fine doing my own baby monitoring but later after I stay so long in labour I was tired just wanted to deliver woman stopped monitoring as was tired |
| 18-28 | The monitoring was good because it helps me to know that my baby was doing fine |
| 18-28 | Declined monitoring – is in too much pain |
| 18-28 | The monitoring was good, I felt fine listening to my babies |
| 18-28 | I felt good doing my own baby monitoring this is a very good thing that has started |
| 29-39 | The monitoring was good, I felt fine doing it |
| 29-39 | The monitor was good from the beginning I felt fine but was tired later because of the pain patient stopped monitoring because she was tired |
| 18-28 | The monitoring was good, I felt fine doing it |
| 29-39 | The monitoring was good. I felt fine knowing that my baby was alright |
| 29-39 | The monitoring was good, it helps me give the power to push my baby |
| 29-39 | The monitoring was good, it made me get closer to my baby |

Additional File 2. Table of all maternal comments on their experience of undertaking monitoring of their unborn babies during labour categorised by age groups.

| Age Group years | Comments from mothers |
|---|---|
| 18-28 | The monitoring was good, I feel fine about it |
| 29-39 | The monitoring was good, I felt fine doing it |
| 29-39 | The monitoring was good, I felt fine listening to my own baby |
| 18-28 | The monitoring was good, it helps me go through my pain for my baby |
| 18-28 | The monitoring was good. Even though it was not easy with the pain but it is good to listen to your own baby |
| 29-39 | The monitoring was good, it helps me go through my pain |
| 18-28 | Patient told me she was so happy for the new introduced fetal monitoring programme and has express interest in doing it |
| 17 and below | The monitor was good it help me with my pain |
| 29-39 | The monitoring was very good because it helps me go through my pain knowing my baby was fine |
| 18-28 | The monitoring was good, I felt fine listening to my baby |
| 18-28 | The monitoring was good. It is fine to listen to your own baby, it makes you feel good |
| 17 and below | It was good, I feel fine |
| 17 and below | The monitoring was good, I felt good listening to my baby |
| N/A | Mother was happy with the monitoring, according to her she was happy to hear her baby heart beat |

Additional File 2. Table of all maternal comments on their experience of undertaking monitoring of their unborn babies during labour categorised by age groups.

| Age Group years | Comments from mothers |
|---|---|
| N/A | Mum said she was happy with the process and enjoy listening to her baby heart |
| N/A | Patient feel comfortable listening to her baby heart |
| N/A | Mother was happy listening to her baby FHT. According to her she felt part of her care |
| N/A | Mother express her interest in measuring her baby FHR. She further stated due to the exercise she will always come to NAME OF HOSPITAL for maternity care during pregnancy |
| N/A | I felt that I am important when you told me to be a part of my baby monitoring process. It helps me a lot |
| N/A | Mother felt comfortable using this method. She told me that she will encourage her friends who have not been seeking care at NAME OF HOSPITAL because of the fetal monitoring (illegible). The only problem was locating the FHT on her abdomen |
| 29-39 | The monitoring was good. It help even us that cannot read or write listen to our own baby |
| N/A | Mother felt happy with this new method of monitoring. She promised to give birth at this facility any time she is pregnant because of this new programme. There was no problem |
| N/A | Mother was happy listening to her baby heart beat. According to her she felt part of her care. The only problem was locating the FHT on her abdomen |
| N/A | I thank God for the programme I am happy to hear my baby heart beat. Please continue it |
| N/A | I enjoy doing it good, thank you doctor |
| N/A | I am happy about this, it help listen to my baby heart |
| N/A | I am comfortable doing this as it helped me form part of my baby monitoring |
| N/A | Thank you, doctor, making me hear my baby breathing. It was very good, I hope to do it always |

Additional File 2. Table of all maternal comments on their experience of undertaking monitoring of their unborn babies during labour categorised by age groups.

| Age Group years | Comments from mothers |
|---|---|
| N/A | It was very good to do what the doctor say to do in order to listen to my baby heart beat |
| 29-39 | According to patient she lost her fetus during past pregnancy. Here she was happy when she noticed her fetal heart beat was dropping and the quick response that was processed |
| N/A | This program is good. It has help me listen to my baby heart. I am so happy |
| N/A | According to the patient she was happy to use a modern equipment to listen to the baby. She said she will spread the information to her friends |
| N/A | Thank doctor for making me hear my baby heart |
| N/A | **Ultrasound confirmed fetal death before monitoring started** |
| N/A | I enjoy doing it with you to listen to my baby heart sound |
| N/A | I enjoy it very great thank God for your help |
| 29-39 | Good. |
| 29-39 | I feel good in monitoring my baby in my stomach |
| 18-28 | I feel very good looking after my baby |
| 29-39 | It was helpful to me. No problem. thank you |
| 29-39 | Thank you for making me to listen to my baby. I will always come to this hospital. I don't have problem with this process. It is helpful. Thanks again |
| 17 and below | Thank you for helping me monitor my baby. I am happy. There was no problem. |

Additional File 2. Table of all maternal comments on their experience of undertaking monitoring of their unborn babies during labour categorised by age groups.

| Age Group years | Comments from mothers |
|---|---|
| 18-28 | Listening to my baby heart sound was very helpful to me. I felt that my right was respected as I took in my baby monitoring. Thanks for this program. I am happy. |
| 18-28 | I am happy to hear my baby heart. I knew that I was carrying a live baby in my womb. |
| 18-28 | According to patient, she was happy with the process and express her desire to continue seeking care at NAME OF HOSPITAL anytime she is pregnant |
| 29-39 | This new thing (here?) might people bring good. You look to your own child. Is very good |
| 29-39 | (Main) complaint that this procedure is helpful but when in labour, labour pain cannot permit for her to do this |
| 18-28 | It was good but it is uncomfortable |
| 18-28 | According to patient, she find it a good thing but the pain is there. It can be uncomfortable to do. |
| 17 and below | Thank you for this. It help me but I was in pain and so it make me angery first but I overcome it later |
| 18-28 | It is good to watch my baby |
| 18-28 | Patient reported that doing this is good. She felt happy about this. She said there was no problem. |
| 18-28 | According to her, the monitoring was good but had problem with locate the site due to the labor pain |
| 29-39 | Patient says that she likes this process, but the only problem is the discomfort she experienced during contractions and (find) the site of the FHT |
| 18-28 | Good according to the patient. |
| 18-28 | According to her it was good no problem |

Additional File 2. Table of all maternal comments on their experience of undertaking monitoring of their unborn babies during labour categorised by age groups.

| Age Group years | Comments from mothers |
| --- | --- |
| 29-39 | Good according to the patient |
| 18-28 | (unclear) Gives me joy and makes me feel part of my health. (unclear) I am grateful (unclear) problem |
| 18-28 | I thank you for this process. It was good. I don't have any problem with that patient said |
| 18-28 | I am happy. No problem. |
| 29-39 | I am grateful for this process. No problems |
| 18-28 | I am happy with this. I do not have any problem. |
| 18-28 | I "thank" you for this process. I like it. Nothing wrong with it |
| 29-39 | She inform me that it was fine to listen to her fetus heart tone (sound). There was no problem with them |
| 18-28 | It was good. No problem thanks to you |
| 17 and below | This new method of monitoring was helpful to me and my baby. No problems for doing this |
| 18-28 | Patient inform me that the process is good but labor is uncomfortable and so doing it is disturbing |
| 18-28 | She said it was good to listen to her fetus and reported no problem |
| 18-28 | After the process and birth the patient express her happiness but complain at same time during contraction it can be uncomfortable to do |
| 18-28 | According to patient, loves the process but the labor pain can be embarrassing in the process |

Additional File 2. Table of all maternal comments on their experience of undertaking monitoring of their unborn babies during labour categorised by age groups.

| Age Group years | Comments from mothers |
|---|---|
| 18-28 | I am feeling grateful to God for taking care of my baby. It was good |
| 18-28 | you for this process. It good. Thank you. |
| 18-28 | I feeling happy over this new method of baby monitoring. It help me a lot. It is just labor stress that seems embarrassing in doing it |
| 29-39 | Getting involved in the process is something amazing to me. I felt part of my care and thank God that I have a live baby |
| 18-28 | After a safe delivery, patient inform me that the process was good but during labor pains it can be uncomfortable |
| 18-28 | I am happy for this process to help me listen to my baby heart no problem. |
| 18-28 | Thanks to God Almighty for this process it helps me a lot but the pain can distress. |
| 17 and below | Listening to my baby heart was good. It help me to know that something was happening to him. No problem with it. Thank you. |
| 18-28 | The heartbeat of my child during labor was very great. Not really any problem was experienced. |
| 17 and below | It felt good listening to my baby. No problem |
| 18-28 | I like the thing I was doing but it was hard to do because of the pain. |
| 18-28 | According to patient" I happy to monitor my baby heart tone. I not get any problem with it |
| 18-28 | It was good doing this. I am happy about It but labor pain can be to disturbing and stress also but never mind it was not bad |
| 18-28 | It was helpful to me. There was no problem |

Additional File 2. Table of all maternal comments on their experience of undertaking monitoring of their unborn babies during labour categorised by age groups.

| Age Group years | Comments from mothers |
|---|---|
| 29-39 | I am happy being part of my baby monitoring no problem with it thank you |
| 40+ | Thank you for it but the pain can be too much |
| 29-39 | That my first time to do this thing but I was hearing my heart beating but my stomach was just hurting that it was making it hard |
| 29-39 | It was not bad but when your stomach hurting you can not do anything because of the pain |
| 29-39 | Thank you. No problem |
| 18-28 | It was alright Nothing wrong listen to my baby heart but the pain was giving me a hard time |
| 18-28 | I am thankful for this but the pain is too much during the process |
| 18-28 | My people you thank you for the thing oh but the pain can be too much that all |
| 18-28 | It alright but my stomach was hurting too much so it was giving me a hard time |
| 18-28 | Thank you for making me hear by baby breathing |
| 18-28 | Monitoring my baby heart was good. It just has some discomfort in doing it due to the stomach pain but I was encourage because of the help. |
| 29-39 | "It not bad thing for me to listen to my baby heart sound but the pain can give me hard time so to do it can be hard (bad?)" |
| 18-28 | Thank you for the process. It was difficulty to do but it is good. |
| 18-28 | "I was hearing my baby heart sound. It make me happy because I hadn't do it before but the pain can be too much" |

Additional File 2. Table of all maternal comments on their experience of undertaking monitoring of their unborn babies during labour categorised by age groups.

| Age Group years | Comments from mothers |
|---|---|
| 18-28 | Thank you for the process. It is really helpful listening to my baby heart the only discomfort is during the pain you don't (want?) to do it, it is not bad |
| 29-39 | That good thing to hear my baby heart sound your thank you but the pain was given me hard time. |
| 29-39 | Your thank you I was listening to my ownself baby by myself but when the pain ready to come it can be small thing that the only problem I get with it. |
| 18-28 | It was good listening to my baby heart. It make me feel important, but the only problem is the discomfort from the pain. |
| 18-28 | it not bad for me to listen myself but the pain can make people can want to do anything self. |
| 18-28 | It alright to be listening to my own baby heart sound but the pain can be too much that all. |
| 18-28 | I was not vex. First the time I start but the time the pain was getting severe I not wanted to do it but since you talk to me to do it that how it do it. But it not bad thing. It good. |
| 29-39 | Your thank you for helping me to listen to my own baby heart sound. The pain can give people hard time that all. |
| 18-28 | The pain can hurt but it was not bad thing to listen to my ownself baby heart. The only thing the pain can be too much that all. |
| 29-39 | Your thank you for making to listen to my baby heart the time I was in pain. The only problem that the pain can give people hard time. |
| 18-28 | It is a good thing to monitor my baby. It was good but the discomfort from the labor pain can be embarrassing. |
| 18-28 | It alright to listen to my baby heart sound only the pain was too much that all. |
| 18-28 | It not bad thing you thank you. But the pain can make you (you can) want to do it that all. |
| 18-28 | Thank you for this program. If not so my baby was going to die. The only thing that the pain. |

Additional File 2. Table of all maternal comments on their experience of undertaking monitoring of their unborn babies during labour categorised by age groups.

| Age Group years | Comments from mothers |
|---|---|
| 18-28 | No problem. It alright to listen to my baby heart sound. |
| 18-28 | Thank you for saving my life and my baby. It really helpful to listen to my baby heart to know what was happening to me. |
| 18-28 | I feel good when I was listening to my baby heart. It help me to know what happen to my baby. |
| 18-28 | Thank you doctor for making to hear my baby sounds |
| 18-28 | I very much today (thank?) God to hear my baby breathing |
| 18-28 | For me I got to show that I will do this again because I can be tire |
| 18-28 | I feel very good today that my first time to do this. |
| 29-39 | It was very good to hear my baby heart beats |
| 18-28 | Please I not got anything to say |
| 40+ | I like it very much. It help me to hear my baby. |
| 18-28 | This is really good. I will tell my friends that still pregnant to (be?) delivered at the hospital. |
| 18-28 | I enjoy it so much before the government was not doing it. I hope you will continue |
| 17 and below | I find it good it was Ok but the pain tummy when I put the machine on my stomach to hear my baby I feel happy. No problem |
| 18-28 | I not got anything to say to you just to say thank you and the white people that bring this |

Additional File 2. Table of all maternal comments on their experience of undertaking monitoring of their unborn babies during labour categorised by age groups.

| **Age Group years** | **Comments from mothers** |
|---|---|
| 40+ | It was good to do it but that the pain was giving me hard time. |
| 17 and below | Your thank you for the thing xxx I know listening to my ownself baby myself the only problem that the pain was too much |
| 18-28 | It was not bad I like the way it was looking it Ok but the pain too much |
| 18-28 | I am cool with the self baby monitoring. It is helpful though the labor pain is uncomfortable but on the overall it is OK |
| 18-28 | "You thank you it alright it help me" |
| 29-39 | I feeling pain too much. I will not be able to continue this thing unlike (unless?) the pain come down |
| 18-28 | Thank you so much for your patience but at least I able to hear my baby. My last belly I don't see someone doing it for me |
| 29-39 | It alright the thing you doing but the pain can hurt. It can make it that you not want to do it but it not bad. |
| 18-28 | I feel important in the coming of my baby. This modern method is very important it help a lot thank you |
| 18-28 | Obs clinician/doctor called because patient detected low FHT during her monitoring. |
| 18-28 | I like it so much doctor that real good thing the government put in place here. I will tell all my sisters that pregnant to come to the hospital |
| 18-28 | I was feeling pain the time was checking my heart but lastly I started enjoying it. |
| 18-28 | Thank you doctor for making me to hear my baby breathing on the machine. I really enjoy it. |
| 18-28 | Patient was very happy, according to patient this procedure is fine, it helps you know bad sound before time |

Additional File 2. Table of all maternal comments on their experience of undertaking monitoring of their unborn babies during labour categorised by age groups.

| Age Group years | Comments from mothers |
|---|---|
| 18 -28 | Patient said it was hard to do from the beginning, it is good thing to do, the procedure save her baby |
| 29-39 | according to mum it is a good step to do because it helps you to notice danger sooner |
| 18-28 | It was good not bad. It help me a lot make me know about my baby. |
| 18-28 | Patient declined but we monitoring and she gave birth, later patient apologized that it was a good thing to do, because of the pain she was looking at it as a bad thing |
| 18-28 | Patient started and later said she was tired and feeling weak. And later I took it and started doing myself. Ended with good result |
| 18-28 | Patient later declined to monitor her FHT. Says she was tired of monitoring. She was counsel but still declined |
| 18-28 | I felt good listening to my baby it helped me to go through my pain |
| N/A | It was good. It make me feel good to listen to my own baby |
| 29-39 | It was good it helps me feel good about my baby |
| 18-28 | It was fine. It make me feel good to do new thing |
| 18-28 | It was good because it help me to know about my baby |
| 40+ | It was good. I enjoy doing it. |
| 18-28 | It was good I felt good monitoring my baby heart rate |
| 29-39 | I found the monitoring helpful it helps me go through my pain |

Additional File 2. Table of all maternal comments on their experience of undertaking monitoring of their unborn babies during labour categorised by age groups.

| Age Group years | Comments from mothers |
|---|---|
| 18-28 | I felt good listening to my baby it helped me to learn a new thing |
| 18-28 | It was good listening to my baby. It help me to be strong |
| 18-28 | I found the monitoring very good it helped me to know that my baby was fine |
| 18-28 | It was good make me feel fine to listen to my baby |
| 18-28 | It was good to listen to my baby it helps me know how my baby was doing |
| 18-28 | It was good because it help me to know my baby was OK |
| 18-28 | Monitoring my baby heartbeat make me to know how my baby was doing during labour |
| 17 and below | I feel good about the monitoring it feel good to listen to your baby |
| 18-28 | The monitoring was good make me feel close to my baby |
| 18-28 | The monitoring was good because it help me listen to my own baby |
| 29-39 | The monitoring was good feels good to know that your baby is fine during labour |
| 18-28 | It was very good it made me feel good to listen to my baby |
| 18-28 | It was good doing my fetal heart rate it helped me know about my baby |
| 18-28 | Monitoring was good it help me to know that my baby was fine |

Additional File 2. Table of all maternal comments on their experience of undertaking monitoring of their unborn babies during labour categorised by age groups.

| Age Group years | Comments from mothers |
|---|---|
| 29-39 | It was good I feel fine listening to my baby |
| 18-28 | It was good I felt good listening to my baby |
| 29-39 | It was good and very helpful it makes me feel closer to my baby |
| 18-28 | Monitoring my baby was good |
| 29-39 | Patient declined said she does not want to do fetal monitoring |
| 18-28 | It was good monitoring my own fetal heart rate during labour |
| 18-28 | The monitoring was good I was happy at the beginning but later I stopped because I was feeling bad that I could not deliver on time |
| N/A | The monitoring was good but it is difficult when you are in severe pain |
| 29-39 | The monitoring was good I enjoy doing it |
| 29-39 | It was good I feel fine listening to my baby |
| 18-28 | Yes, monitoring my fetal heart rate was good and I feel good to listen to my baby |
| 18-28 | It was good listening to my baby myself and knowing my baby was fine |
| 18-28 | It was good monitoring my baby even though it was my first time but enjoy doing it |
| 18-28 | It was good I feel good listening to my baby |

Additional File 2. Table of all maternal comments on their experience of undertaking monitoring of their unborn babies during labour categorised by age groups.

| Age Group years | Comments from mothers |
|---|---|
| 18-28 | I feel good listening to my baby make me feel fine my baby is alright |
| 18-28 | Patient declined but we monitor and she gave birth later patient apologise that it is a good thing to do because of the pain she was looking at it to be a bad thing |
| 17 and below | Patient said why doing she felt bad but after she gave birth she felt fine because it helps you to know the danger sign and good sign about your baby |
| 18-28 | Patient started and later said she was tired and feeling weak and later I took it and started doing myself ended with good result |
| 18-28 | patient felt bad from the start but later on after she'd given birth it was a good thing to do because you know whether the baby is alive or dead |
| 29-39 | Patient declined from the beginning but later talk to and at the end she was happy she said it is a good thing to do because make you to take action quick when the baby is not breathing good |
| 18-28 | According to patient she felt very bad from the beginning but later on she felt fine because it helps you to know whether your baby will live or die |
| 17 and below | According to mum this procedure is fine but is not easy to do it if you don't understand you will make confusion |
| 18-28 | According to patient she enjoy doing it because it make her baby to be alive |
| 17 and below | Patient declined from the beginning and after I counselled her she started doing it again and when she was about to give birth she said she was tired and she left it. After she gave birth she said it was good because it mate you to know whether your baby is alive or about to die |
| 29-39 | According to patient she look at it as embarrassment but later on she was feeling happy because there is a good outcome |
| 18-28 | According to patient she found it difficult but it is a good thing to do |
| N/A | According to patient she was encouraged and impressed because she was doing it and not wanting to do it but later on she had a good outcome so she was happy |
| 29-39 | According to mum it was not a easy task to do but she's happy because her baby is fine |

Additional File 2. Table of all maternal comments on their experience of undertaking monitoring of their unborn babies during labour categorised by age groups.

| Age Group years | Comments from mothers |
|---|---|
| 18-28 | Patient declined to do it but I keep doing it until she gave birth she also said it is a good thing to do because of the pain she declined to do it |
| N/A | Patient had a laceration have to clean her was transferred to ward and I asked her how was it and she said it was not easy thank God she had her baby |
| N/A | Patient was happy because it is good to come to hospital you be able to listen to your baby heartbeat and will know that the baby is okay |
| N/A | According to patient is a good thing to do because of the pain is not easy but now she's happy because her baby is alive and she also alive so it's a good thing to do |
| N/A | Patient declined procedure, counsel her in the process and later she started doing it alone. At the end she was sorry for what happen it was because of the pain |
| 29-39 | Patient was very happy last because from the beginning she was not interested later on she told me thank you and said it was the pain |
| 18-28 | According to mum she was not feeling fine from the beginning because of the pain it is a good thing to do it helps you know about your baby and well-being of the child |
| 18-28 | According to mum, this is the first time seeing patient to be working for her self,she said it is a good thing to do but when in labour is bad because of the pain |
| 18-28 | According to patient she declined it from the beginning she was feeling pain but after I talk to her and she was understood and we began the process and everything went well |
| 18-28 | According to mum monitoring is hard at setting time she knew her babies heart rate was low and we took quick action and now the baby is in her hands so she thank the organisation. |
| 18-28 | According to patient everything we did for her is good let God bless us |
| 18-28 | According to patient everything was alright |
| 17 and below | According to mum it is okay because this help the doctor nurses to take quick action |
| 29-39 | According to patient she's okay with it she tell God thanks for her baby and her health |

Additional File 2. Table of all maternal comments on their experience of undertaking monitoring of their unborn babies during labour categorised by age groups.

| **Age Group years** | **Comments from mothers** |
|---|---|
| 29-39 | Patient explain that listening to the baby heart rate it you do that bad thing is Coming to happen ( death) |
| 17 and below | Patient initially declined procedure but later on she was encouraged to do it herself and everything went well |
| 18-28 | Patient said everything was okay she then thank everybody that is doing this good work |
| 18-28 | Patient said she's very happy because she seen baby breathing well and she has self okay according to patient any time she pregnant she will come and give birth to NAME OF HOSPITAL |
| 29-39 | patient said it was hard to do she appreciated everybody for the hard work |
| 29-39 | according to patient at the beginning she was worried and she was going to do it later she found out that it is a good thing to do |
| N/A | according to patient she was not happy from the beginning because of the pain but it is a good thing to do it helps you know about your baby well-being |
| 18-28 | Patient was very vexed from the beginning but as time went by we kept talking with her and she was able to do it and she start doing it and we ended the monitoring together with her and she was happy |
| 29-39 | According to mum she love the procedure but is not easy to go through |
| 18-28 | According to mum she love the idea because other pregnant women goes to the hospital and comes back with no baby in their hands it looks sorryfull |
| 18-28 | Patient was happy to have the C/S when she saw her baby alive and said it was a good thing to do |
| N/A | According to mum her first time to see  patient seeing herself doing her baby FHT. It's good but not easy to do |
| 28-38 | According to pt every care that went on was fine |
| 18-28 | Pt appreciate the process |

Additional File 2. Table of all maternal comments on their experience of undertaking monitoring of their unborn babies during labour categorised by age groups.

| Age Group years | Comments from mothers |
|---|---|
| 18-28 | Mother complaint about the process but she said it was good her baby to be monitor in labour because she continues to hear her baby until birth |
| 18-28 | Mother commended the process |
| 18-28 | Patient worry when the heart rate was reducing but at last she was happy because her baby came through |
| 18-28 | The mother was interesting in the monitoring, she found it helpful. Patient said it help her know her baby heart beat |
| 18-28 | The mother appreciates the process and thanks the midwife for the process |
| 18-28 | Mother said she found the monitoring helpful in that she has a live baby. She was cooperative and was asking other mothers to join the process. The only problem she has with it is the pain (contraction) during and after, but it is a good thing |
| N/A | According to the patient the monitoring is good but she won't be able to do it. She is tired, she is feeling pain. She wont want to do anything. |
| 29-39 | Mother said she was happy with the monitoring because she could have had a dead baby if she didn't monitor. She's also asking other mothers to accept and be part of the process |
| 18-28 | Mum was not happy from the beginning but later on she started doing it small |
| 29-39 | Patient was happy with the procedure and she appreciated us for the work |
| N/A | Mother was very cooperative; she was interesting and said it helped her to deliver a live male infant. She monitor from start of labor to end |
| 29-39 | Patient declined from the beginning but later started doing it and it went well. She welcome the idea |
| 17 and below | Patient was very happy because she call for help and action was taken quickly by the OB clinician and her baby was save |
| 18-28 | Patient agree to work along with us until she gave birth safely. She was happy with the idea |

Additional File 2. Table of all maternal comments on their experience of undertaking monitoring of their unborn babies during labour categorised by age groups.

| Age Group years | Comments from mothers |
|---|---|
| N/A | According to she was very happy and she told everybody thanks because of the monitoring her baby was saved |
| 18-28 | Mother expressed interest in monitoring her own baby and appreciate us for educating her how pregnant can monitor her baby during delivery to help detect any problem |
| 17 and below | Pt admitted that it was good thing for herself to listen to her baby heart beat. It made her believed that her baby can breathe inside her mother womb |
| N/A | Mother said it was her first time to hear that baby can breathe in the womb. She like it was fine for her to monitor or listen to her own baby heart beat |
| 18-28 | Mother said the monitoring help her with her baby, she got a live baby. She was willing and cooperative and ask other mothers to accept the monitoring |
| 29-39 | She said the process is help but the pain make her confuse and she was having interest and enjoy hearing her baby until she gave birth |
| 18-28 | Patient was very happy because of the outcome |
| 29-39 | Patient enjoy the procedure and appreciate the effort |
| 29-39 | Mother appreciated the procedure and thanks the program |
| 17 and below | Mother was happy to hear her baby heart beat because she stay in labour for long and worry about her unborn baby |
| N/A | Mother she consent and started monitoring as she was even happy hearing her own baby. She was interesting and said it help her by giving her a live baby |
| N/A | Mother said she likes the monitoring, she agreed to monitor but cant do it herself because of the pain |
| N/A | According to mother monitoring is good but she cannot continue it herself due to pain. At last she said it help her with a live neonate |
| N/A | Patient first declined monitoring, She was counselled on the importance of the monitoring regardless of educational status. Pt later accepted and found it helpful. She said it help her have a live baby |

Additional File 2. Table of all maternal comments on their experience of undertaking monitoring of their unborn babies during labour categorised by age groups.

| Age Group years | Comments from mothers |
|---|---|
| N/A | Mother was interested in the monitoring, according to her it help her have a live baby. |
| N/A | She said it help her greatly by having a live neonate. She was also concern about knowing more about the doppler, FHR normal and abnormal range. She was informed. |
| N/A | According to mother the monitoring is good, it help her deliver her baby live. She was interested in doing it |
| N/A | Mother agreed to the process, she started it but discontinue due to pain and was helped by midwife and OB clinician. Mother said it's a good thing, it help her have a live baby |
| 17 and below | Mother admitted that she like the program |
| 18-28 | Appreciated the listening to her baby until birth. She recommended that all laboring mothers should be able to listen to their fetus during labour |
| 18-28 | According to patient she was surprised to know that baby heart can beat in the mother stomach and it help her to know about her baby wellbeing |
| 29-39 | Mother expressed happiness in helping to monitor her baby wellbeing before birth. She thanks the midwives for helping her |
| 18-28 | It was good for me to be involved in giving care to my unborn child. A big thanks to you all for helping me |
| 18-28 | It was good to help manage her baby in her stomach. She has been hearing that baby can breathe in the stomach but today she got to know that the baby heart can beat in the stomach |
| N/A | I feel good about it and also want for other mother or pregnant woman to do same |
| 29-39 | This patient is a nurse herself, monitoring her baby contractions until she deliver her baby, and she it is a good idea |
| 18-28 | Mom appreciate listening to her fetal heart rate until she give birth |
| 29-39 | The monitoring was good, it is a good idea and I hope it will continue because it will save a lot of babies as it did mine. Sometimes the midwives are busy so this will help them, and help us the mothers too |

Additional File 2. Table of all maternal comments on their experience of undertaking monitoring of their unborn babies during labour categorised by age groups.

| Age Group years | Comments from mothers |
|---|---|
| 29-39 | Mother complained that the labour pain can make it difficult to do the FHR. But it was good listening to my babe |
| 17 and below | Good listening to my own baby, make me feel encouraged to go through my labour |
| 29-39 | Yes it helps her be sure of baby breathing |
| 18-28 | This prime appreciated the listening to her baby until birth because she was happy to get live baby. It's the grace of God. |
| 18-28 | Mother felt fine monitoring her baby, she said it is a good idea |
| 29-39 | Patient said it was good to be involved in the monitoring of her baby in her stomach |
| 29-39 | According to mum hard but I felt fine monitoring my baby |
| 17 and below | According to mum she felt fine monitoring her baby |
| 18-28 | According to mum she enjoyed monitoring her baby heart rate |
| 18-28 | The pain was too much but I was happy to listen to my baby heart beat |
| 18-28 | I appreciate hearing my baby until I born my baby. I like to have the same chance to listen to my unborn baby the next time I am in labour |
| 18-28 | I am happy that my listen to baby heart beat |
| 17 and below | It was very painful but I was happy to listen to my baby heart beat |
| 29-39 | I enjoy listening to my baby but my next labour there should be pain medicine for labour |

Additional File 2. Table of all maternal comments on their experience of undertaking monitoring of their unborn babies during labour categorised by age groups.

| Age Group years | Comments from mothers |
|---|---|
| 18-28 | according to mum she felt good monitoring her baby |
| 29-39 | Thanks to God for this program because listening to my baby make me strong to work for my baby and myself |
| 18-28 | it was so good to hear my baby heart beat. Thank you for teaching me |
| 18-28 | I give many thanks to the program for listening to my baby encouraged me to bear with the labour pain |
| 17 and below | I felt fine listening to my baby |
| 18-28 | I felt very good monitoring my baby it was a good thing to help monitor my baby |
| 29-39 | I felt fine monitoring my baby |
| 18-28 | the woman was happy to listen to her baby heart beat |
| 29-39 | this woman was very happy to listen to her baby heart beat throughout the process |
| 18-28 | But is very much happy to listen to my baby heart beat |
| 18-28 | patient expressed herself of happiness in listening to her baby heart beating before delivery. Thankyou for making to know new thing |
| 29-39 | listening to my baby was good.is my first time seeing big belly doing her own baby monitoring |
| 18-28 | I feel happy listening to my own baby heart beat. This monitoring help me to have a live baby |
| 17 and below | it was too good for me to be involved in monitoring my baby heart beating |

Additional File 2. Table of all maternal comments on their experience of undertaking monitoring of their unborn babies during labour categorised by age groups.

| Age Group years | Comments from mothers |
|---|---|
| 18-28 | I like to have the same means of listening to my own baby anytime I am pregnant again |
| 29-39 | I appreciate the process of monitoring my baby until I born. It was good for me because I stay long in labour but I was still hearing my baby which made me happier |
| 18-28 | this mother cannot read or write but she is happy to be listening to her baby heart beat |
| 29-39 | It help because with all the pain I refused to listen to them. I still got my baby by talking to me good. I found it very good because it help me in getting my baby. No problem. |
| 29-39 | It was fine because the baby breathing well. It help me because my baby was born alive. No problem. |
| 18-28 | I feel fine in helping to monitor my baby heartbeat. It help me to know if my baby is still alive and it do not give me any problem except when my stomach was hurting very hard |
| 18-28 | It was alright. It help to know whether the child is breath good. No problem |
| 17 and below | It felt fine monitoring my baby but is really not easy. It helps me because my baby is alive no problem |
| 18-28 | I was feeling fine monitoring my baby. Yes it help me because my baby is alive. No problem. |
| 18-28 | I was fine while monitoring my baby. It help me because my baby is alive. No problem |
| 29-39 | It was fine thanks God. I was happy in monitoring my baby heart beat. |
| 18-28 | Everything was fine. Thank God |
| 18-28 | I felt happy when I was monitoring my own baby |
| 29-39 | I like monitoring. It make me born a live baby. |

Additional File 2. Table of all maternal comments on their experience of undertaking monitoring of their unborn babies during labour categorised by age groups.

| Age Group years | Comments from mothers |
|---|---|
| 29-39 | I like listening to my baby heart but I don't know if my baby will live again now that I am going to a different hospital |
| 29-39 | I Like the monitoring. I was happy listening to my baby. The monitoring help me to have a live baby. |
| 40+ | Listening to my baby was good. I like it, it is hard to be in pain and listen to your baby but it is good. Listening to my baby make me to have a living baby. |
| 18-28 | I like the monitoring. I enjoy listening to my baby even though he didn't survive |
| N/A | The monitoring was alright for me. It help me to put more effort for my baby. To know that my baby is still living in my stomach. |
| 17 and below | I see the monitoring good for me and my baby because it my make me to know that my baby is still living. |
| 29-39 | It is hard to be in pain and monitor your baby. You must be doing it for us. Thank God my baby is living but it is too hard. The machine can cause more pain on the stomach. |
| 18-28 | I like the monitoring. It make me born a living baby but it is hard to do. It is hard to be in pain and holding the machine. |
| N/A | The monitoring is good but it is hard. Thank God it make my baby to come out live but it is hard to be holding the machine in your hand while in pain |
| 18-28 | I was happy with the monitoring. I like it but it is too hard. Thank God I born my baby alive. Your thank you for helping me. It was not easy. |
| 18-28 | To monitor my baby is good but it can hurt me when I am doing it. Thank God I have a live baby. |
| 18-28 | I like the monitoring but it can be hard to do. It help me born a living baby. No problem |
| 17 and below | The monitoring is good. It help me in that I delivered a live baby. It did not cause any problem on me. Patient says she is in the 4$^{th}$ grade. |
| 40+ | I feel fine monitoring my baby. It help me because after all the monitoring Caesarean section was done and I still have my baby. No problem |

Additional File 2. Table of all maternal comments on their experience of undertaking monitoring of their unborn babies during labour categorised by age groups.

| Age Group years | Comments from mothers |
|---|---|
| 29-39 | It was so good. Did not give me any problem only when my stomach was hurting hard. Thank you for helping me. |
| 18-28 | I find the monitoring good but not easy to do. It helps me and my baby because baby is in good condition. No problem. |
| 18-28 | I like listening to my baby because my baby is OK. Yes it helps me because my baby is OK. No it did not cause any problem for me and my baby |
| 29-39 | I feel fine monitoring my baby. It help me and no problem at all |
| 29-39 | It help me because it did not allow me to go to surgery. It help me because my baby was born alive and by normal vaginal delivery. It help me so much even though ….is more difficult to do but I try doing to have got good result |
| 18-28 | A help because my baby was born alive and in good condition. A help me but very difficult but I try doing it and heal a good result. No problem at all. |
| 18-28 | I enjoy listening to my baby but the program should get small medicine for stomach hurting time to born baby |
| 18-28 | Very much happy. It was not bad to do because it help or make me to know about my baby |
| 18-28 | Midwife took over. Mother was uncomfortable to continue due to severe uterine contractions between 9 and 10 cm dilatation<br>It was too helpful. Helps me to monitor my children in my stomach. Thank |
| 17 and below | Thank you for helping me it was too helpful |
| 18-28 | It was very good. It helps me to know about my baby. Did not cause any problem. |
| 18-28 | It was okay and did not cause me any harm. Thank very much |
| 18-28 | Thank you very much the monitoring help me to know that I was carrying a baby whose heart was beating. It was my first time to know |
| 29-39 | It was very very helpful and not bad to do. Thank you |

Additional File 2. Table of all maternal comments on their experience of undertaking monitoring of their unborn babies during labour categorised by age groups.

| Age Group years | Comments from mothers |
|---|---|
| 18-28 | I am very happy to be included in monitoring my baby heart beating. It was not too hard. Thank you |
| 29-39 | Very good and thank you. It helps me a lot did not cause me any problem |
| 18-28 | Due to severe uterine contractions when cervix reached 10 cm dilated midwife took over<br>It was good it helps me monitor my baby. It was uncomfortable for me when my stomach was hurting too hard. Thank you very much. |
| 18-28 | It was good for my baby and myself because the monitoring make me to have hope for my baby life. I thank God for the program. |
| 17 and below | I felt fine. It help me and my baby because my baby is alive. It help me when I was in labour because my baby is alive. No problem at all. |
| 18-28 | It is good fine and didn't cause me any harm. Thank you very much. |
| 18-28 | Thank God it was too fine. No it did not cause me any problems. Thank you |
| 18-28 | The monitoring is good but I was not able to do it all by myself because of the pain and my foot pain. Yes my baby is living so it help. No problem with it but the pain can be too much. |
| 18-28 | The monitor help me to inform the midwife that my baby was not breathing good. So I see it to be good for all the big belly with stomach hurting pain. |
| 18-28 | It is very good and helpful to me. At least all big belays should know how to do the monitoring before the stomach can hurt. |
| 29-39 | The patient only ask the team on call to help her do the marking . She said because of the pain she unable to hold pen<br>I like the monitoring it make my baby live. No problem with the monitoring. It only hard to hold the machine when your stomach hurting. |
| 29-39 | I feel good monitoring my baby. Yes it help me greatly because my baby is alive. No problem at all. |
| 18-28 | It is good and did not cause any problem |
| 29-39 | The monitoring was good. Yes it help with my labour. It make me know that the FHT was good. No it did not cause me any problems.<br>Patient wrote for herself. She's a registered midwife by profession. |

Additional File 2. Table of all maternal comments on their experience of undertaking monitoring of their unborn babies during labour categorised by age groups.

| Age Group years | Comments from mothers |
|---|---|
| 17 and below | The monitoring help me to know that my babies are two in my stomach. I tell the programme thank you for coming to us |
| 17 and below | This is good. I like the monitoring. It help my labour pain. I born living baby. I see no problem with it. |
| 29-39 | This is very good idea. I like the monitoring. I see no problem with it |
| 18-28 | I feel fine with the monitoring and yes it helped me because I have to see my baby alive |
| 29-39 | I NAME REMOVED find the monitoring good by I myself listening to it too. No it did not cause me any problem |
| 29-39 | I find the monitoring good. It help me greatly. No problem at all. |
| 18-28 | It was fine. There was no problem. Thank you |
| 18-28 G1 P0 | I find it good. It help me because my baby is alive. No problem with it. |
| 40 + | According to patient everything we did for her was okay |
| 18-28 | Very good. Did (not) cause me any problem. Thank you for teaching and ? Me in the monitoring of my baby |
| 18-28 | It was not bad and I feel good doing it. Thank you for helping me to monitor my own baby |
| 18-28 | Very fine thank you |
| 18-28 | Fine no problem with the process. Thank you very much |
| 18-28 | The monitoring help to keep I and my baby alive, yes it help with my labor. By the grace of God there's no problem. |

Additional File 2. Table of all maternal comments on their experience of undertaking monitoring of their unborn babies during labour categorised by age groups.

| Age Group years | Comments from mothers |
|---|---|
| 18-28 | The monitoring was good because I had my child alive. Yes it help me a lot to know the FH of the child. No problems caused for me |
| 18-28 | I like the way I hear my baby until born. I tell the programme thanks you. |
| 18-28 | I feel fine listening to my baby, yes help me so much in that my baby is alive, no problem at all |
| 18-28 | I like the way I listen to my baby until I deliver. It help me so much in that my baby is alive no problem |
| 17 and below | I enjoy hearing my baby until born. Thank God for the people who sent the maternal (material?) for me to listen to my baby |
| 17 and below | The listening to my baby make me strong to be active until born my baby. |
| 18-28 | Thanks you it help me very much to know about my baby |
| 29-39 | It was fine and did not give me any problem. It help me to monitor my baby too. Thanks you very much. |
| 18-28 | The monitoring is alright. I like it. It help me to know that my baby was alive. It never cause me any problem. |
| 18-28 | I feel good doing the baby monitoring. It make me born my baby alive. No problem with the monitoring. |
| 18-28 | The monitoring is good for me. Yes it help me to born my baby. No problem with the monitoring |
| 18-28 | I feel good about the monitoring because it save my baby life. Yes it help me in that baby. No problem |
| 18-28 | *Patient only did the monitoring one time. OB clinician and midwife completed the monitoring until she gave birth.* Patient said the pain is too much. She is tired. |
| 18-28 | Thank you for the baby monitoring. It was good. No problem with it. |

Additional File 2. Table of all maternal comments on their experience of undertaking monitoring of their unborn babies during labour categorised by age groups.

| Age Group years | Comments from mothers |
|---|---|
| 17 and below | Patient agreed (to do the monitoring) at first but later she declined after doing it for a while. She said the pain is too much for her. The sonicaid was painful for her |
| 29-39 | It was very good did not give me any problem. It help me to know about my baby while he was in my stomach. Thank you. |
| 17 and below | It was too good and no problem was encountered |
| 29-39 | Did not give me any problem. It was very fine thank you. |
| 29-39 | I see the monitoring to be good. No problem it cause me. I have a live baby |
| 29-39 | I find the monitoring good. Yes it help me greatly. It help me and my baby the both of us are alive. No problem |
| 18-28 | I find the monitoring good because I was worry about the position of my baby and by the grace of God my baby born alive. Yes it help me |
| 29-39 | I like the monitoring. It is good for me to make me born my baby alive. There was no problem |
| 29-39 | It very fine not bad thank you to everyone of you for helping me |
| 18-28 | Thank God it was not bad or hard. It make me to know how to help monitor my baby. Thank you. |
| 29-39 | It was not bad for me. It help me a lot to get involved in monitoring my own baby. Thank you. |
| 18-28 | Thank you for the monitoring. It was good. I didn’t have any problem doing it. |
| 18-28 | Very much okay. Not bad. It also makes me to know about my unborn child. Thank you very much for helping me. |

NA = Not available/Not collected
